# On closed‐shell interactions between heavy main‐group elements

**DOI:** 10.1002/jcc.26999

**Published:** 2022-09-21

**Authors:** Lars Kloo

**Affiliations:** ^1^ Applied Physical Chemistry, Department of Chemistry KTH Royal Institute of Technology Stockholm Sweden

**Keywords:** closed‐shell interaction, covalent interaction, intermolecular interactions, main‐group elements

## Abstract

A series of di‐ and polymetal complexes involving closed‐shell, heavy main‐group atoms and ions shows a selection of special physical properties. These involve short metal–metal contacts, low entropies of formation and, most interestingly, strong Raman bands at low wavenumbers. These results together with the constitution of the coordination compounds, where the majority of electrons are assembled on the highly polarizable metal atoms and ions, experimental results have been interpreted in terms of direct, partial covalent metal–metal bonding. Previous theoretical studies have challenged this view and instead attributed the obvious attractive forces involved to secondary‐type of interactions, such as dispersion. This study utilizes a multitude of theoretical tools, such as natural bond order (NBO) and natural energy decomposition analysis (NEDA), non‐covalent interaction (NCI) analysis, electron localization functions (ELFs), and atoms‐in‐molecules (AIM) to characterize the interactions in models comprising closed‐shell dimers, as well as experimentally studied ring and cage systems constituting the main reason for the hypotheses on metal–metal interactions. The results show that all experimental results can be attributed to the covalent interactions between the electron‐rich, metal centers and the bridging anions in ring and cage coordination compounds of high symmetry, where the experimentally observed effects can be traced to the combination of covalent interactions between the metal centers and the anions along the edges of the ring and cage systems in combination with the cooperative effects generated by the high symmetry of these ring and cage systems.

## Graphical abstract

Polymetal complexes based on closed‐shell atoms involve short metal–metal contacts and strong Raman bands. These have caused claims of direct, covalent metal–metal bonding. The current study utilizes a multitude of theoretical tools to analyze the type of interactions. The results show that the observed effects can be attributed to covalent interactions along the edges of the systems, rather than direct metal–metal bonding, in combination with the high symmetry of these ring and cage systems.

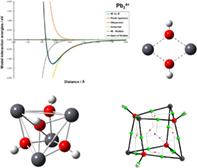

## 1 INTRODUCTION

Based on experimental indicators, such as short bridged and non‐bridged atom–atom contacts, acute angles over bridging ligands and spectroscopic features, in particular from vibrational spectroscopy at low wavenumbers, many compounds in the literature on the chemistry of heavy main‐group elements (mainly from the periods 5 and 6) include suggestions of covalent, closed‐shell interactions. Several examples can be found among the polymetal hydrolysis products of In(III), Sn(II), Pb(I), and Bi(III),^1^ such as In_4_(OH)_6_^6+^, Sn_3_(OH)_4_^2+^, Pb_4_(OH)_4_^4+^, and Bi_6_O_4_(OH)_4_^6+^ to mention a few species analogous to the more well‐known Tl_4_(OR)_4_ alkoxide compounds (R = small alkyl group),^2^ but also among di‐ and polymetal species in the iodide systems,^3^ such as Ag_4_I^3+^ and Hg_2_I^3+^. This is also the background for the current theoretical investigation of such interactions.

The area is vast and versatile, and therefore this study will concentrate on dimetal complexes stabilized in molten nitrate salts, as well as chemically and structurally highly related systems.^4^ Several dimetal complexes, M_2_X^*m*+^, of the heavy main‐group metals were investigated, and in many of these systems, an unexpected thermodynamic pattern in terms of negative changes in the entropy of formation combined with short M–M contacts could be observed. This type of coordination compounds can be regarded as an inverse of the classical Werner complexes characterized by a metal ion as coordination center surrounded by ligands.^5^ In terms of acid–base theory, the metal‐ion coordination centers act as Lewis acids and the ligands as Lewis bases. The dimetal compounds are instead based on a Lewis base as coordination center and Lewis acids, that is, positively charged metal ions, as ligands. This is a very common building block in main‐group solid compounds and are, at least up to the early 1990s, thoroughly reviewed.^4^ In particular, the systems based on lead(II), a closed‐shell ligand with [Xe]4f^14^5d^10^6s^2^ electron configuration, showed negative entropy changes of formation combined with short metal ion contacts.^6^ A subsequent theoretical investigation at extended‐Hückel level indicated that these experimentally observed features could be interpreted as originating from partial covalent metal–metal interactions at the Pb—Pb distances detected.^7^

Because of the extensive broadening of the Rayleigh shoulder in the Raman spectra from nitrate melts, it is difficult to extract any spectroscopic information at low wavenumbers. However, Brooker and coworkers could identify strong signals at around 140 cm^−1^ in molten PbO–PbSO_4_ mixtures in the anti‐Stokes region,^8^ as well as in molten PbO–Pb(NO_3_)_2_–(K,Na)NO_3_ systems using depolarized Raman spectroscopy.^9^ These signals show that a symmetrical stretch vibration in the molecular constituents of the molten mixtures involve a significant change in polarizability and suggest that covalent interactions need to be considered. It is logical to attribute such a change in polarizability to involving an orbital overlap of the very electron‐rich lead(II) ions, even if regarded as a relatively hard ion in the oxidation state +II taking the hard‐soft, acid–base formalism into account; viz. in terms of Pb—Pb stretch vibrations. These spectroscopic results are further verified below regarding highly analogous systems among the lead(II) hydrolysis products in aqueous solution.

Several studies of the hydrolysis of Pb(II) in aqueous solution in an ionic medium indicate the formation of di‐ and multilead species, such as Pb_2_OH^3+^, Pb_2_(OH)_2_^2+^, Pb_4_(OH)_4_^4+^, and Pb_6_(OH)_8_^4+^ ^10^; the latter system later reformulated to Pb_6_O(OH)_6_^4+^ on the basis of liquid structural studies.^11^ A speciation analysis shows Pb_4_(OH)_4_^4+^ to be predominant species over a rather larger compositional range, thus being available for detailed vibrational and structural analyses.^12^ Pb_4_(OH)_4_^4+^ was shown to have an approximate cubane structure with the Pb(II) ions in the corners of a tetrahedron with Pb—Pb distances of about 3.85 Å. In contemporary Raman spectroscopic studies of Pb_4_(OH)_4_^4+^ in solution, and in isolated solids, Maroni and Spiro noted several low‐wavenumber bands. In particular, the bands in the range 130–140 cm^−1^ were highlighted and taken as an indication of direct Pb—Pb bonding.^13^ Although indicated in the text of the cited papers, a crystal structure of Pb_4_(OH)_4_^4+^ was first published in 1974 reporting Pb—Pb distances ranging 3.72–3.95 Å.^1c^ This class of compounds have more recently found a re‐newed interest because of environmental issues and optical properties, and new crystal structures in combination with different anions have been reported.^14^ Another, and related system of closed‐shell interactions with interesting optical properties involve Au(I)–Pb(II) interactions, although these interactions were on the basis of theoretical computations attributed to dispersion and ionic interactions.^15^ Some related studies may be mentioned in this context, where very short Pb(II)‐Pb(II) contacts of 3.19 Å were observed in Pb_2_Si_5_N_8_,^16^ and even 2.85 Å in oxo/hydroxo‐bridged Pb_4_ clusters confined in zeolites.^17^ However, a theoretical study using the hybrid density‐functional B3LYP showed the Pb—Pb interaction in Pb_4_(OH)_4_^4+^ to be weakly antibonding; in contrast to implications from experimental structural and spectroscopic results.^18^ Instead, the importance of the Pb^2+^–OH^−^ interactions was promoted.

The cubane‐type of Pb_4_(OH)_4_^4+^ cage shows both structural and compositional similarities with the Tl‐alkoxide compounds Tl_4_(OR)_4_ (R = Me, Et, or *n*‐Pr), and it is not surprising that early work on the two separate types of systems to a large extent was pursued by the same research groups. Spiro and coworker published a series of structural and spectroscopic studies on both the lead(II) hydroxo cage compounds, as well as on the thallium(I) alkoxo cage compounds. The Tl(I) alkoxides were known since the 1860s, but the crystal structure of Tl_4_(OMe)_4_ was not reported until about 100 years later.^2b^ The structure was described in terms of a distorted cube with Tl—O—Tl angles >90°. The intramolecular Tl—Tl distances range 3.81–3.86 Å, and it is interesting to note that the intercubane Tl—Tl distances only are about 0.2 Å longer. A later study on a series of thallium(I) alkoxides reported one analogous cubane structure for Tl_4_(OCH_2_CMe_3_)_4_ with Tl—O distances of 2.46–2.47 Å and Tl—Tl distances of 3.75–3.77 Å, thus corresponding to a Tl—O(R)—Tl angle of about 100°.^19^ A few years later than the initial structural report, Maroni and Spiro investigated the Raman‐spectroscopic properties of this type of Tl‐containing cubane structures and noted low‐wavenumber bands clearly adhering to the cubane or tetrahedral organization of the atoms; in particular the Tl atoms.^20^ A band at 102 cm^−1^ was attributed to primarily a symmetrical Tl—Tl stretching mode, and it was concluded that Tl—Tl interaction was present albeit weaker than in the analogous lead(II) structure of Pb_4_(OH)_4_^4+^. The main conclusions were summarized and discussed in comparison to similar compounds by Spiro in a 1970 review.^2a^ In this context, it is notable that our own studies on the Tl^+^–Br^−^ system in molten nitrate media indeed showed the formation of a bromide‐bridged Tl_2_Br^+^ species, although these, in contrast to the corresponding Pb^2+^–Br^−^ system, did not show the signatures of direct Tl—Tl bonding in terms of acute Tl—Br—Tl angles and a negative change of formation entropy.^21^

The results from the structural and spectroscopic studies of the Pb(II) and Tl(I) systems described above suggest some sort of direct metal–metal interaction, in spite of the metal ions formally being closed‐shell with no nonpaired electrons available for obvious covalent interaction. In a very comprehensive review, Pyykkö analyzed closed‐shell interactions in a large number of compounds, in particular the cases of d^10^–d^10^ and s^2^–s^2^ interactions.^22^ Pyykkö noted that the heavy main‐groups elements, albeit closed‐shell, appear to be “sticky.” He more specifically highlighted that no advanced theoretical methods had been used to investigate the Pb(II) and Tl(I) systems, and that bridging anions may play a major role. In general terms, Pyykkö attributed the effects observed experimentally to correlation and dispersion enhanced by relativistic effects. This definitely casts another light on the conclusions from experiment and suggests that other effects can be at play in the closed‐shell Pb(II) and Tl(I) systems.

The systems investigated in this work of course bear resemblance to the in many papers discussed d^10^–d^10^ interactions, especially for the coinage metal +I ions and in particular for the Au(I) ions, in terms of aurophilicity. However, in spite of the analogies, the closed‐shell d^10^–d^10^ interactions will not be in focus in the present study. In view of the conflicting results from experiment and theory regarding the Pb(II) and Tl(I) systems, the most puzzling result is the strong low‐wavenumber Raman signals observed. Can these emerge in other ways than involving the large and soft electron clouds of the heavy main‐group metals? The ambition of the current study is to use a wide range of theoretical models as tools to gain a deeper insight into the interaction scheme in the systems discussed above and aims to clarify the nature of the predominant interactions involved.

## 2 METHODS AND COMPUTATIONAL DETAILS

All quantum chemical calculations were performed using Gaussian16 (Rev. B.01 and C.01)^23^ and Turbomole 7.2.^24^ ECP‐based basis sets were used for Au, Hg, Tl, Pb, and Bi, employing the Stuttgart‐Dresden‐Cologne groups, relativistic effective core potentials (ECPs; MDF60 for all period‐6 elements, MDF28 for I and MDF10 for Br) and valence spaces of (14s11p10d3f2g1h)/[6s6p5d3f2g1h] quality for Au and Hg,^25^ (14s11p11d2f1g)/[6s6p4d2f1g] for Tl,^26^ (14s11p11d2f1g)/[6s6p4d2f1g] for Pb and Bi,^26^, ^27^ (14s11p12d2f1g)[6s6p4d2f1g] for I,^28^ and (14s11p12d2f1g)/[6s5p4d2f1g] for Br.^29^ Basis sets for H, N, O, and Cl were of 6‐311G quality with amended polarization and diffuse functions [6‐311G++(3df,2pd) keyword in Gaussian]. The corresponding basis sets were used in Turbomole, including the 2‐component versions of the MDF ECPs. Calculations were performed at second‐order Møller–Plesset perturbation theory (MP2) level and at density‐functional level (DFT) using the B97‐D functional including the Grimme dispersion corrections.^30^ Slightly truncated basis sets for Tl, O, C, and H were used in the calculations of the Raman bands and associated activities for the computationally large Tl_4_(OEt)_4_ system at MP2 level to allow results in a reasonable time.

Analyses of the computed molecular orbitals were made using the AIMALL,^31^ the Multiwfn programs,^32^ as well as the natural bond order (NBO) and natural energy decomposition analysis (NEDA) functionalities as interfaced with NBO 7.0 in Gaussian 16.^33^ Non‐covalent interaction (NCI) analyses were made on the basis of wavefunctions generated from the calculations at MP2 level, whereas the NEDA analyses were made at DFT level. Isosurfaces were visualized using VMD 1.9 And GaussView 6.^34^

## 3 RESULTS AND DISCUSSION

### 3.1 M–M potential energy surfaces and insights into the interatomic interaction

This section will focus on the interaction profile of a series of closed‐shell, almost isoelectronic [M–M]^*m*+/−^ pairs, where M = Au, Hg, Tl, Pb, and Bi from the Period 6 in the periodic table all represent a valence electron configuration of [Xe]4f^14^5d^10^ (Au^−^ and Hg) and [Xe]4f^14^5d^10^6s^2^ (Tl, Pb, and Bi). All except the Hg_2_ pair will be charged, and therefore it is clear already from the start that electrostatic repulsion will dominate the interaction between the charged atoms. However, because of the effects discussed in the introductory section, the main focus will be to extract information regarding attractive interactions hidden below the fog of electrostatic repulsion and what dominates such an attraction, if existing. The main reason for including a series of systems with different, and zero, charge is to extract general knowledge extending beyond the specifics of a single system.

**FIGURE 1:**
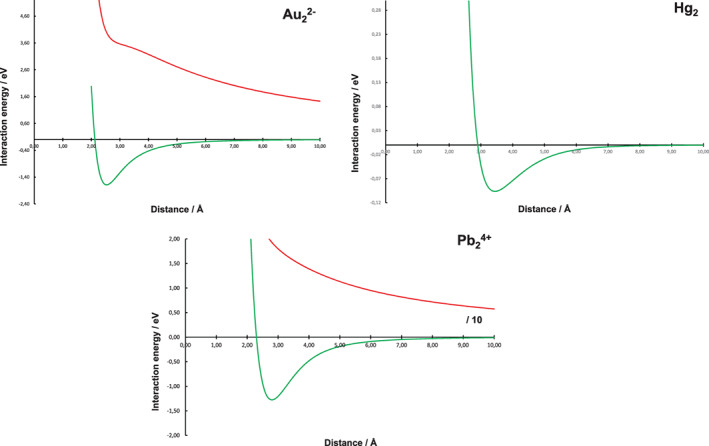
The PESs of three representative systems at MP2‐level for the binuclear systems including (red) and excluding (green) direct electrostatic repulsion. The results for all systems are shown in the Supporting Information

Figure 1 shows the potential energy surfaces (PESs) of three representative binuclear systems, including and excluding the direct electrostatic repulsion (the results from all five systems are shown in Figure S1). The electrostatic repulsion is modeled by simply subtracting the Coulombic energy for two point charges positioned at the interatomic distance.

It is clear that all the heavy main‐group atoms excerpt some sort of attraction underneath the direct electrostatic repulsion. The PES shapes are like typical two‐atom interaction patterns, and they are characterized by a minimum distance of approximately 2.55 Å for Au_2_^2−^ (−1.71 eV), 3.50 Å for Hg_2_ (−0.10 eV), 3.20 Å for Tl_2_^2+^ (−0.33 eV), 2.80 Å for Pb_2_^4+^ (−1.25 eV), and 2.50 Å for Bi_2_^6+^ (−3.05 eV). The potential‐well depths are given within parentheses above, to be compared to the Coulombic repulsive energies for the −2‐charged Au_2_^2−^ of −5.65 eV, +2‐charged Tl_2_^2+^ of −4.50 eV, the +4‐charged Pb_2_^4+^ of −20.6 eV and the +6‐charged Bi_2_^6+^ of a massive −51.8 eV. We can note two phenomena extracted from the minimum distances. First, the minimum distance is the longest for the neutral Hg_2_ dimer, which may give a hint to the types of interaction involved. The charged dimers are likely to experience interactions including secondary electrostatics, such as induction, to a higher degree than the neutral Hg_2_ species thus accounting for the shorter minimum distances. Second, the observed correlation between the higher positive charge and shorter minimum distances points in the same direction. These expectations are to part verified by an analysis of *d* ln(*E*)/*d* ln*R* relationship, where *E* denotes the interaction energy and *R* the interatomic distance, arriving at 6 for Hg_2_ system and at 4 for all the charged M_2_ systems (see Figure S3). This indicates a 1/*R* ^6^ dependence of the interaction energy for the Hg_2_ system, suggesting dispersion to be the dominant type of interaction, and a 1/*R* ^4^ dependence for the charged systems, instead indicating induction to be predominant. In order to gain a deeper insight into the interaction scheme in the five dimeric systems, simple models of different types can be applied to the PESs. By building complexity from the most simple and expected types of interactions, we will learn what is needed to model the PESs and thereby what types of interactions that, alongside direct electrostatic repulsion, will account for the “stickiness” of the heavy, closed‐shell atoms observed by Pyykkö. The simple models applied are discussed in some more detail in the Supporting Information.

**FIGURE 2:**
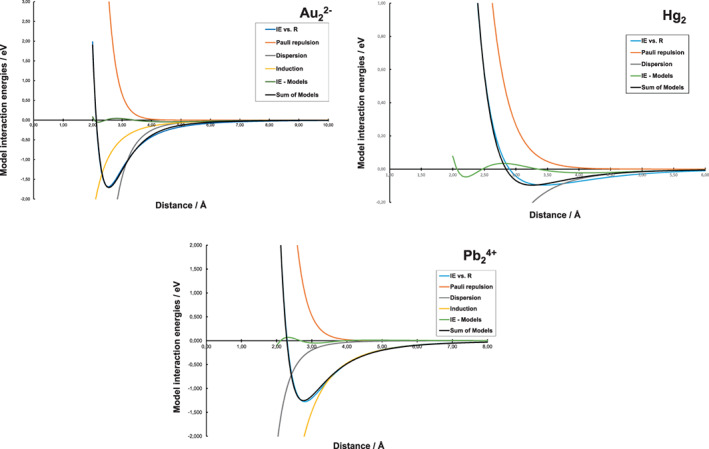
Models applied to the PESs at MP2‐level for the three representative binuclear systems excluding direct electrostatic repulsion. The results for all systems are shown in the Supporting Information

Figure 2 shows the results of modeling in three representative systems (results for all five systems are shown in Figure S2). Starting with the system of least complexity, the neutral Hg_2_ dimer, we note that inclusion of Pauli repulsion and dispersion are fully sufficient to model the PES. This simple model was fitted to the computational PES using a least‐squares approach, and there are minor deviations at short Hg‐Hg distances. However, the simple model can be regarded as sufficient to describe the repulsive and attractive forces emerging as the potential well in the PES of Hg_2_. Thus, the Hg_2_ dimer is bound through mainly dispersion with a dissociation energy just below 0.1 eV. Overall, the results obtained are in good agreement with previously reported advanced computational studies on the Hg_2_ dimer.^35^

The most closely related system, because they are isoelectronic, is the Au_2_^2−^ dimer. Also in this system, inclusion of Pauli repulsion and dispersion offers a relatively good model for the Au^−^–Au^−^ interaction. Addition of induction‐type interaction shows a slight improvement in the total model, which should not be surprising considering the negative charge of the gold ions in spite of the overall low charge density. Going to the systems also including a valence 6s^2^ electron pair and positive charge, we start with the Tl_2_^2+^ system that electrostatically closest mimics the Au_2_^2−^ dimer. We can observe a few differences; first, the minimum distance for the Tl_2_^2+^ dimer is longer and the interaction well substantially more shallow (about 5 times with respect to Au_2_^2−^). It is likely that this can be attributed to the difference in valence electron configuration, where Tl^+^ also incorporates a diffuse 6s^2^ electron pair. Second, in the bonding description it becomes more clear that induction is required to properly model the PES in the Tl_2_^2+^ pair, as compared to Au_2_^2−^. Going to Pb_2_^4+^ characterized by even higher direct electrostatic repulsion, the main observation is that induction really becomes the predominant type of secondary interaction and must be included as a part of the interaction model in the Pb_2_^4+^ and Bi_2_^6+^ systems. In conclusion, the models required for the PES of all five M_2_^*m*+/−^ systems indicate that the stickiness observed in heavy main‐group systems involving these elements can be attributed to secondary interaction forces, predominantly to dispersion and induction.

From an orbital interaction point of view, one should expect a similar bonding scheme as in a noble‐gas dimer, where every bonding atomic orbital interaction is outweighed by the corresponding antibonding interaction resulting in a covalently nonbonded system. Mulliken and NBO population analyses of the M_2_^*m*+/−^ dimers at atom–atom distances around the minimum distances, obtained after subtraction of the direct electrostatic effect, give no indication of orbital overlap and direct covalent interaction between the heavy metal atoms in the dimers studied.

In order to get some further insights into the interaction scheme in the systems, the M_2_^*m*+/−^ dimers were exposed to an NCI analysis. In an NCI analysis both the gradient of the electron density and the eigenvalues of the Hessian of the energy matrix are used for characterization of the interaction types in terms of attraction, repulsion, or van der Waal‐type (vdW) of interactions. The resulting isosurfaces are shown in Figure S4. The Hg_2_ and Tl_2_^2+^ systems are dominated by vdW interactions, although some degree of covalent interaction is indicated in the thallium dimer. The charged Au_2_^2−^ and Bi_2_^6+^ systems are clearly dominated by steric (repulsion) effects, but the interaction scheme of the Pb_2_^4+^ unit looks rather complex involving both steric and covalent contributions. In summary, steric repulsion or vdW interactions seem to dominate the picture.

Another tool for characterization focuses on the contribution of different types of interaction to the total interaction energy, NEDA. Table S1 shows the results for the M_2_^*m*+/−^ dimers. Of most relevance to the current systems is the balance between static Coulombic, induced electrostatic and charge‐transfer (covalent type of) interactions. The results in Table S1 essentially mimic the ones observed in Figures 1 and 2. Only Hg_2_ is weakly bonding, and in all other systems Coulombic repulsion dominates. As the total charge increases, also the induced interaction (viz. induction) increases in energy contribution. It is notable that the charge‐transfer contribution is higher than the Coulombic contribution for Au_2_^2−^, but for the cationic systems it only amounts to 11% in the Tl_2_^2+^, 6% in the Pb_2_^4+^ and 4% in the Bi_2_^6+^ system.

The above analysis was made primarily at MP2 level of theory, and it is necessary to make a sensitivity test with respect to both Hamiltonian (or functional) and the inclusion of relativistic effects. Since the neutral and charged species represent two different types of systems with respect to the predominant interactions, as outlined above, the Hg_2_ and Pb_2_^4+^ systems were selected for such a sensitivity analysis. First, the MP2 calculations were complemented by calculations at DFT level using the B97‐D functional, including Grimme dispersion correction and as employed by two different program systems, Gaussian 16 and Turbomole 7.2.^30^ The use of Turbomole also allows more advanced handling of relativistic effects. Second, relativistic effects, including spin‐orbit coupling at the two‐component level, were also investigated. Such effects have recently been shown to be of importance in dimers of the coinage metal elements.^36^ Scalar relativistic effects are already considered using the relativistic ECPs in the basis sets of the heavy elements.

**FIGURE 3:**
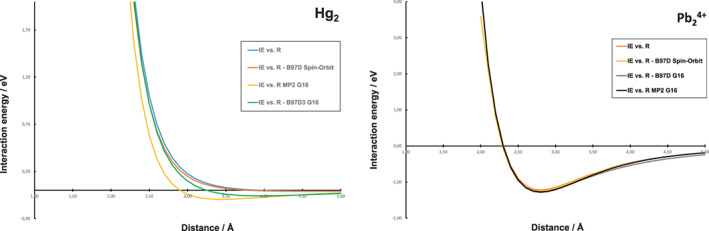
A comparison of computed PESs for Hg_2_ and Pb_2_^4+^ using MP2, DFT, and inclusion of spin‐orbit effects. It should be noted that the direct electrostatic repulsion has been subtracted from the PESs in t Pb_2_^4+^ system, as described in the text.

A comparison between the PESs of Hg_2_ and Pb_2_^4+^ are shown in Figure 3. It is clear that the different types of calculations generate highly similar PESs for the Pb_2_^4+^ system; in particular, the shape of the PESs are quite similar and therefore the analysis performed at MP2 level above should be analogous for an analysis also at DFT level and when explicitly including relativistic spin‐orbit coupling. However, for the neutral Hg_2_ system, where dispersion is predominant, the results deviate more, where the results from the DFT calculations do not fully account for the dispersion interaction suggesting the Hg_2_ unit to be only marginally bound. In spite of this, it is clear that inclusion of spin‐orbit coupling offers no dramatically different results.

### 3.2 Bonding analysis in representative di‐ and polymetal Pb and Tl compounds

In this section, some representative molecular systems studied experimentally are more explicitly analyzed regarding the character of the metal–metal interaction. The main objective here is to investigate if the bonding, typically in a bridging configuration of two metal ions of the type studied above, can induce changes opening for covalent interaction between the metal ions. The main objective is to identify the reasons for the observed Raman intensity of the breathing vibrational modes of this type of compounds. Can there be other explanations than the primary hypothesis including direct covalent metal–metal interaction giving rise to the required vibrationally induced changes in polarizability to generate a Raman signal of significant intensity?

In the models studied computationally, all types of solvation have been neglected except for the bridging species in the form of ligand hydroxide ions or ionic‐liquid solvent anions (nitrate ions). Nonbridging solvation is here presumed to be comparably weak and furthermore to have less impact on any potential metal–metal interaction. In this context it can be noted that the relative permittivity of nitrate melts typically is quite high, in the high‐end range of polar molecular solvents, and thus damping of electrostatic interactions is therefore expected to be significant.^37^

Regarding cationic, dimeric lead systems, the Pb_2_X^3+^ ion, X = F, Cl, Br, and I, in theoretical geometry optimizations all come out as essentially linear compounds, following the expectation from basic Coulombic repulsion between the positively charged lead ions, and no further bonding analysis is necessary for these systems. The main geometrical and vibrational features of the systems investigated are given in Table 1. The Pb_2_I^3+^ system deviates from expectation rendering a nonlinear configuration as the optimal geometry. However, the PES of Pb—I—Pb bending seems to be quite flat at DFT level with a very shallow minimum at around 150° making the optimal angle slightly ambiguous. This seems to be a common feature for the Pb_2_I^3+^ system when applying a wide range of DFT functionals, from historically early variants via more advanced to quite commonly used hybrid functionals. MP2 gives a more well defined and continuously descending PES from 90° to 180° and therefore arrives at a clear 180° energy minimum for the Pb—I—Pb angle in the Pb_2_I^3+^ system.

**TABLE 1:** Selected geometrical and vibrational features of the molecular, bridged Pb(II) systems; results from theory and experiment

		Distance (Å)				Pb—X—Pb angle (°)		Pb—Pb vibration	
		Theory		Experiment		Theory	Experiment	Theory	
System/level	Symmetry	Pb—X (Å)^a^	Pb—Pb (Å)	Pb—X (Å)^a^	Pb—Pb (Å)	Pb—X (Å)^a^	Pb—Pb (Å)	1/*λ* (cm^−1^)	Intensity (Å^4^ amu^−1^)^b^
Pb_2_F^3+^/MP2	C_2v_/D_∞h_	2.281		2.3^c^	3.81	180.0	112		
DFT^d^		2.329				180.0			
Pb_2_Cl^3+^/MP2	C_2v_/D_∞h_	2.685		2.52	3.38	179.9	84		
DFT^d^		2.761				179.8			
Pb_2_Br^3+^/MP2^e^	C_2v_/D_∞h_	2.800		2.61	3.39	179.9	81		
DFT^d^		2.914				180.0			
Pb_2_Br(NO_3_)^2+^/MP2	C_2v_	2.733	3.710			85.5		166	28
(Monodentate)/DFT^d^		2.843	3.987			89.0		174	25
Pb_2_I^3+^/MP2	C_2v_/D_∞h_	2.981				180.0			
DFT^d^		3.142				151.4			
Pb_2_(OH)_2_^2+^/MP2	D_2h_	2.221	3.658	2.4^g^	3.7^g^	110.9	78	130	2.6
DFT^d^		2.290	3.778			111.2		123	4.2
Pb_4_(OH)_4_^4+^/MP2	T_d_	2.435	3.892	2.57^h^	3.83^h^	106.1	96	126^f^ ^,^ ^i^	4.1
DFT^d^		2.527	4.036			106.0		124	7.2

^a^ X = F, Cl, Br, I, O(H).

^b^ The rather odd unit for Raman intensity used in Gaussian16; conversion factor to SI units: 7.41 × 10^−34^.

^c^ Approximate Pb—F distance from liquid x‐ray scattering; taken from reference 6c.

^d^ B97‐D functional.

^e^ A deviating structure, where an O^2−^ ion almost has been ripped off the coordinating nitrate anion.

^f^ The assignment of these vibration modes as the symmetrical breathing mode is not unambiguous.

^g^ Approximate distances taken from different systems based on liquid x‐ray scattering; reference 6d.

^h^ Approximate distances taken from aqueous liquid‐ray scattering; reference 11.

^i^ Experimental polarized Raman band detected at 130 cm^−1^, reference 13.

**FIGURE 4:**
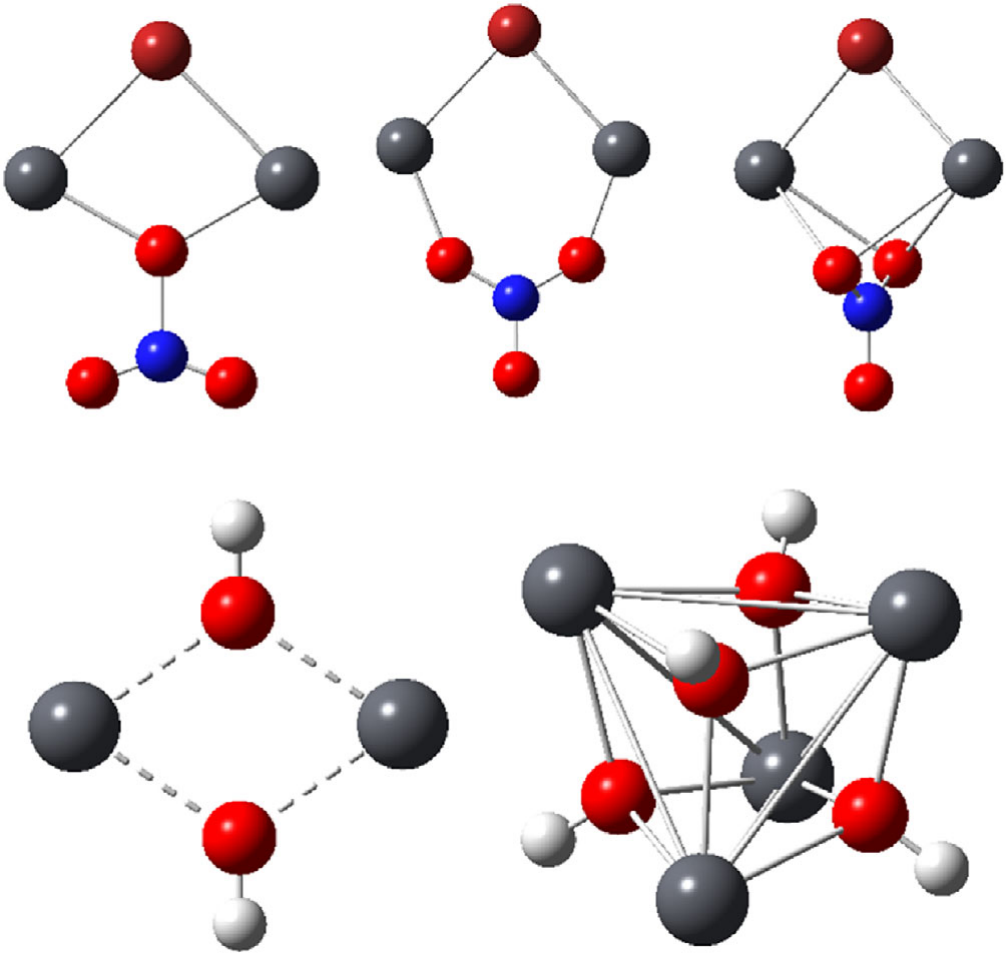
Molecular structures of the Pb(II) ring/cage systems in Table 1, with the three bridging nitrate conformations in the top and the two hydroxide‐bridged systems at the bottom; Br, burgundy; H, white; N, blue; O, red; Pb gray

As indicated experimentally from structural studies of the dimeric lead complexes in molten nitrate media, the experimental results indicate a distinct bridging interaction involving nitrate ions as well. In reference 6c, three models of such a bridging interaction were proposed, see the top of Figure 4. In this study, only the monodentate, bridging model was considered, since it is the simplest and is furthermore most likely to be qualitatively representative for all bridging conformations. The monodentate, bridging Pb—O(NO_2_) distances in this model were obtained as 2.184 Å at MP2 level and 2.303 Å at DFT level.

Metal ions work as Lewis acids allowing negatively charged bridging anions to donate electron density into empty metal atomic orbitals (AOs). This could generate the necessary prerequisites of direct covalent interaction between the heavy main‐group metal ions. Therefore, the ring and cage systems included in Table 1 and Figure 4 were exposed to an analysis based on natural bond orbital (NBO) populations, as well as the Bader atoms‐in‐molecules (AIM) formalism. The results from the NBO population analyses are given in Table 2.

**TABLE 2:** Computed natural bond (NBO) Pb charges and Pb—Pb overlap populations in the above described ring systems

System/level	Symmetry	Pb charge	Pb—Pb Wiberg bond index	Pb—X^a^ Wiberg bond index
Pb_2_Br(NO_3_)^2+^/MP2	C_2v_	+1.55	0.012	0.414
(Monodentate)/DFT^b^		+1.57	0.010	0.484
Pb_2_(OH)_2_^2+^/MP2	D_2h_	+1.75	0.010	0.206
DFT^b^		+1.73	0.013	0.253
Pb_4_(OH)_4_^4+^/MP2	T_d_	+1.76	0.003	0.128
DFT^b^		+1.75	0.003	0.159

^a^ X = F, Cl, Br, I, O(H).

^b^ B97‐D functional.

**FIGURE 5:**
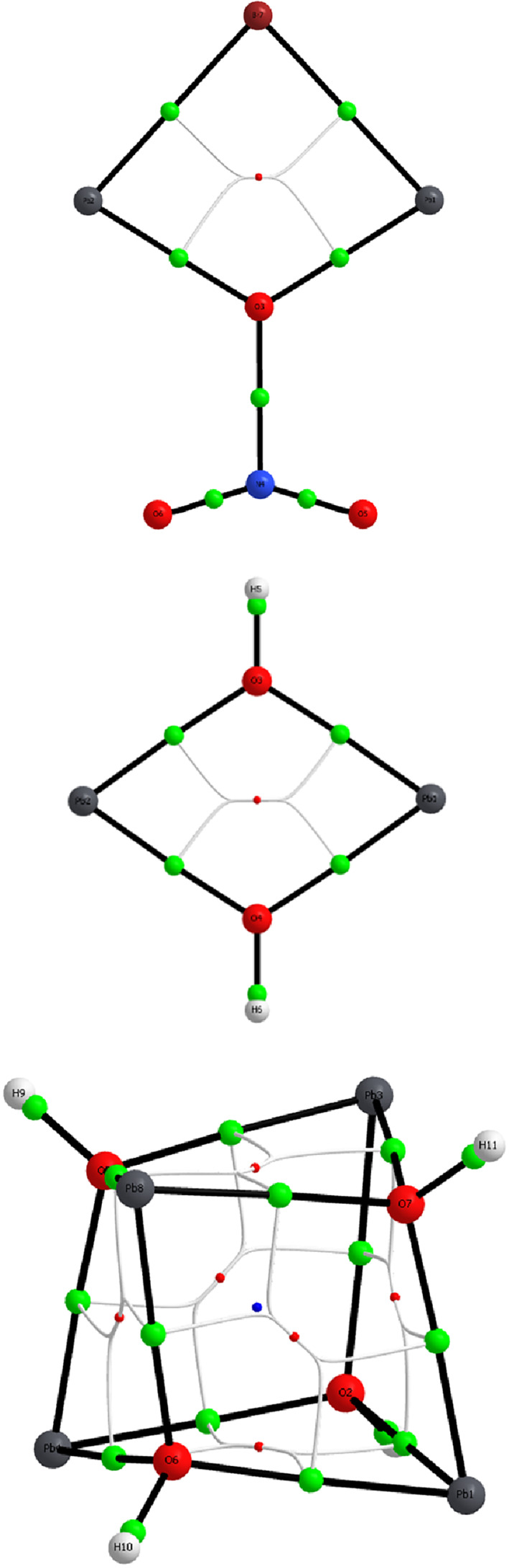
Atoms‐in‐molecules (AIM) images of the Pb(II) systems in order from Table 2 at MP2 level including bond (green), ring (red), and cage (blue) critical points, as well as ring‐to‐bond critical point paths highlighting that covalent bonding only can be observed along the ring/cage edges

The NBO overlap populations in Table 2 clearly show that if there is any M–M orbital interaction in the systems studied, it must be considered insignificant. The bridging anions do not seem to change the conclusions from the “naked” M_2_^*m*+/−^ systems studied above. Application of AIM using the wavefunctions from the computations at MP2 level are shown in Figure 5. It is notable that bond critical points (BCPs) that represent inter‐atomic maxima in electron density can only be identified along the Pb—X (and Pb—O(NO_2_)) bond paths. In the currently studied systems, ring and cage critical points (RCPs, CCPs) represent minima in electron density and emerge because of the covalent bonding along the edges, and therefore these critical points mainly represent a topological consequence of the edge bonding. The results from the AIM analyses essentially support the conclusions from the NBO population analyses with respect to an absence of direct covalent M–M interaction. However, we also get some indications for the cause of the observed Raman signals generated by the systems. That cause can instead be linked to the covalent nature of the M—X and M—O(NO_2_) bonds forming the edges of a ring or cage system of high symmetry. This is well in line with the hypothesis brought forward by Breza and Manova.^18^ Under such circumstances, the cooperative change in polarizability upon molecular ‘breathing’ may be sufficient to generate a strong Raman activity. In this perspective, it is interesting to notice that the symmetrical stretch vibration at MP2 level in Pb_2_(OH)_2_^2+^ associated with the symmetrical breathing of the Pb ions is 130 cm^−1^ with a Raman activity of 3 Å^4^ amu^−1^, whereas the analogous breathing mode involving the hydroxide ions display a mode at 466 cm^−1^ with a Raman activity of 10 Å^4^ amu^−1^. For Pb_4_(OH)_4_^4+^ the corresponding comparison gives the Pb‐related symmetrical vibration mode at 126 cm^−1^ with a Raman activity of 4 Å^4^ amu^−1^, and the corresponding OH^−^‐related vibration mode at 365 cm^−1^ with a Raman activity of 8 Å^4^ amu^−1^. It should be noted that the low‐wavenumber modes described as of Pb—Pb type, of course also involve a significant Pb—O(H) stretching component. The same pattern is obtained in the vibrational analysis at B97‐D level. This further emphasizes that the key to understanding the origin of the Raman activity at low wavenumbers of these and the related Tl(I) systems can be found in the M—O bond stretching in ring or cage systems of high symmetry offering a cooperative effect of coordinated M—O bond stretches.

The NCI method can be regarded as an extension of the AIM method, and the three ring/cage systems in Figure 5 were also exposed to an NCI analysis. The results are shown in Figure S5. The isosurfaces indicate covalent interaction between the lead(II) ions and the bridging ligands, but otherwise steric repulsion is the dominating theme.

**FIGURE 6:**
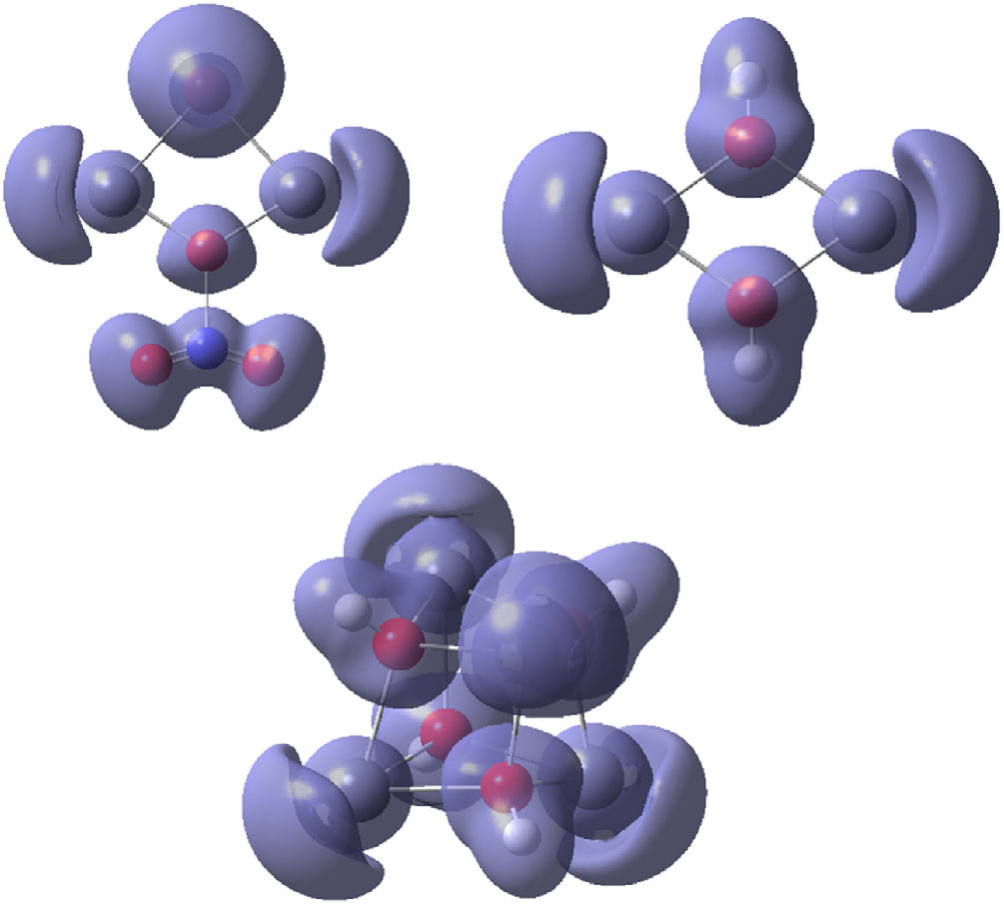
3D electron localization function (ELF) isosurfaces of the Pb(II) systems in order from Table 2 at MP2 level

Another tool of bonding analysis is represented by electron localization functions (ELFs). This method allows visualization of electron‐pair localization in a molecular or extended system, and since covalent bonds represent electron pairing further insights can be obtained from the ELF maps. Therefore, the ring and cage systems represented in Table 2 were also subjected to an ELF analysis. As can be noted from Figure 6, none of the ELF isosurfaces indicate any direct accumulation of electron pair density on the connection vectors between the Pb atoms, in accordance with the results from the NBO‐population and the AIM analyses.

The lead(II)‐containing ring/cage systems can be divided into Pb^2+^, Br^−^, NO_3_^−^, and OH^−^ fragments and the NEDA partitioning into different types of interactions between the fragment be evaluated. As indicated by the NCI isosurfaces, there is a significant degree of covalent interaction between the lead(II) ions and the bridging ligands, although direct and induced electrostatics dominate the contributions to the interaction energy.

**FIGURE 7:**
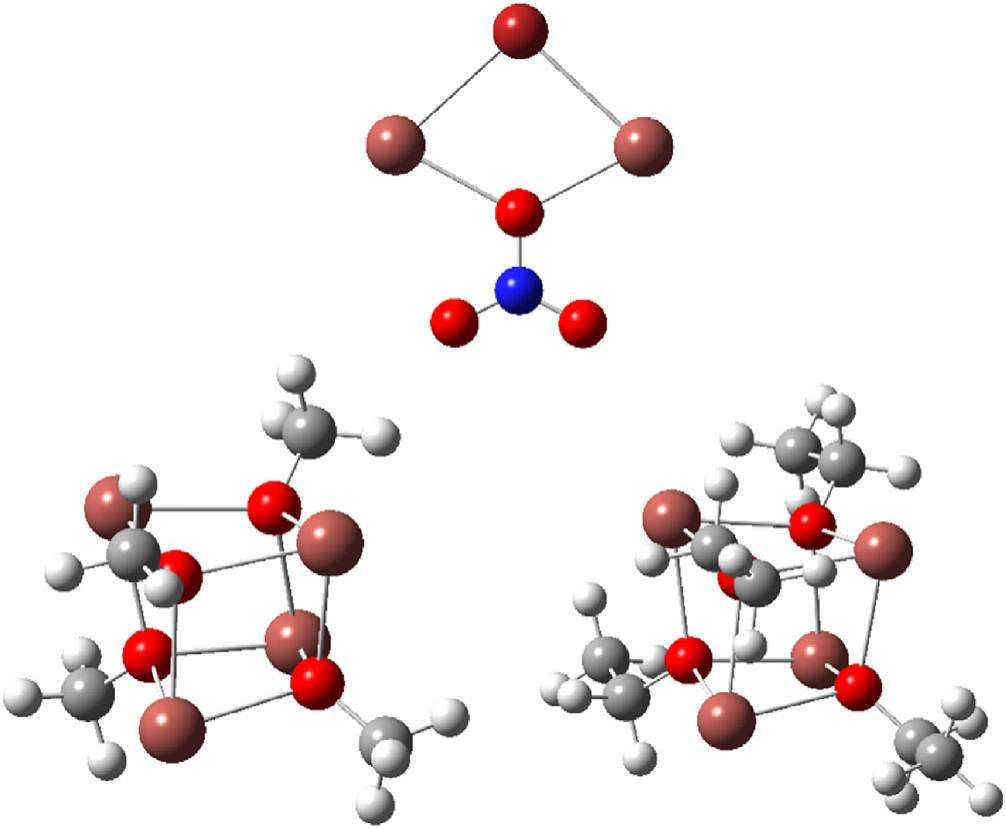
Molecular structures of the Tl(I) ring/cage systems in Table 3. Br, burgundy; C, gray; H, white; N, blue; O, red; Tl, light brown

As noted in the introduction, there is a strong relation between the hydroxo‐bridged Pb(II)‐ion systems and the cage structures of Tl(I) alkoxide compounds regarding both structural and spectroscopic properties. In these systems, the Raman activity of symmetrical, low‐wavenumber vibrational modes come out quite strong being consistent with a tetrahedral Tl_4_ configuration. The totally symmetrical stretch mode (A_1_) dominated by a Tl—Tl stretch emerge at 102 cm^−1^ (the corresponding stretch mode described as of OR^−^ character is registered at 290 cm^−1^ in the Tl_4_(OR)_4_ cages (R = Et and *n*‐Pr)).^2^, ^20^ The lower wavenumbers noted for the Tl_4_‐cage systems, as compared to Pb_4_(OH)_4_^4+^, were interpreted in terms of a weaker M—O bond attributed to the lower polarizing power of a monocation than a dication. The low‐wavenumber bands were for obvious reasons interpreted in terms of direct covalent Tl(I)—Tl(I) bonding. Therefore, these are exposed to an analogous bonding analysis as the Pb(II) systems above, and the Tl_2_Br^+^ dimetal complex is included as a link to the lead(II) systems, although neither the thermodynamics of formation of nor the Tl—Tl distances observed experimentally for the Tl_2_Br^+^ system indicate any direct Tl(I)—Tl(I) interaction. The structures of the model systems studied are shown in Figure 7.

Table 3 summarizes the computed structural and Raman‐spectroscopic results at MP2 and DFT levels, together with the results of an NBO population analysis in Table 4.

**TABLE 3:** Selected geometrical and vibrational features of the molecular, bridged Tl(I) systems; results from theory and experiment

		Distance (Å)						Tl—Tl vibration	
		Theory		Experiment		Tl—X—Tl angle (°)		Theory	
System/level	Symmetry	Tl—X (Å)^a^	Tl—Tl (Å)	Tl—X (Å)^a^	Tl—Tl (Å)	Theory	Experiment	1/*λ* (cm^−1^)	Intensity (Å^4^ amu^−1^)^b^
Tl_2_Br^+^/MP2	C_2v_/D_∞h_	2.810		3.16^d^	4.54^d^	180.0	92		
DFT^d^		2.918				180.0			
Tl_2_Br(NO_3_)/MP2	C_2v_	2.886	4.239			94.5		152	2
(Monodentate)/DFT^c^		2.992	4.322			92.5		137	4
Tl_4_(OMe)_2_/MP2	C_2v_	2.410	3.760	–	3.84^g^	102.5	>90	112^e^	0.3
DFT^c^		2.550	3.947			101.4		111^e^	1.2
Tl_4_(OEt)_4_/MP2	C_1_	2.412	3.729			101.2		106	1.7
DFT^c^		2.568	3.980			101.6		105^f^	4.2

^a^ X = Br, O(Me), O(Et).

^b^ The rather odd unit for Raman intensity used in Gaussian16; conversion factor to SI units: 7.41 × 10^−34^.

^c^ B97‐D functional.

^d^ Taken from reference 21.

^e^ Mainly symmetrical stretch mode of the Tl atoms; OMe group stretch mode is found at 227 cm^−1^ with 11 Å^4^ amu^−1^ Raman activity (MP2); 210 cm^−1^ and Raman activity of 18 Å^4^ amu^−1^ (DFT).

^f^ Same comment as in footnote e; 170 cm^−1^ and a Raman activity of 7.4 Å^4^ amu^−1^ (MP2); 182 cm^−1^ and a Raman activity of 14 Å^4^ amu^−1^ (DFT).

^g^ Taken from reference 2b.

**TABLE 4:** Computed natural bond (NBO) Tl(I) charges and Tl—Tl overlap populations in the above described ring systems

System/level^a^	Symmetry	Tl charge	Tl—Tl Wiberg bond index	Tl—X^b^ Wiberg bond index
Tl_2_Br(NO_3_)/MP2	C_2v_	+0.80	0.003	0.208
(Monodentate)/DFT^c^		+0.79	0.003	0.258
Tl_4_(OMe)_4_/MP2	C_2v_	+0.85	0.048	0.078
DFT^c^		+0.85	0.044	0.090
Tl_4_(OEt)_4_/MP2	C_1_	+0.85	0.068	0.073
DFT^c^		+0.83	0.056	0.087

^a^ The Tl_4_‐cage systems deviate from perfect T_d_ symmetry and the values given are the most.

^b^ X = F, Cl, Br, I, O(H).

^c^ B97‐D functional representative ones.

The results in Tables 3 and 4 clearly show that direct covalent Tl(I)—Tl(I) interaction must be regarded as insignificant, and at the same time there is a clear covalent Tl—O(R) bonding component. Experimental results are scarce but in general agree well with the computed ones.

The importance of covalent interactions in bridging M—L bonds was suggested on the basis of electrochemical electrode, charge‐transfer rates studied by Fronaeus. Dinuclear species of the type M_2_L involving soft M metal ions show considerably higher transfer rates than the corresponding ML monomers, which was interpreted in terms of covalent M—L bonding in the dinuclear species.^38^

**FIGURE 8:**
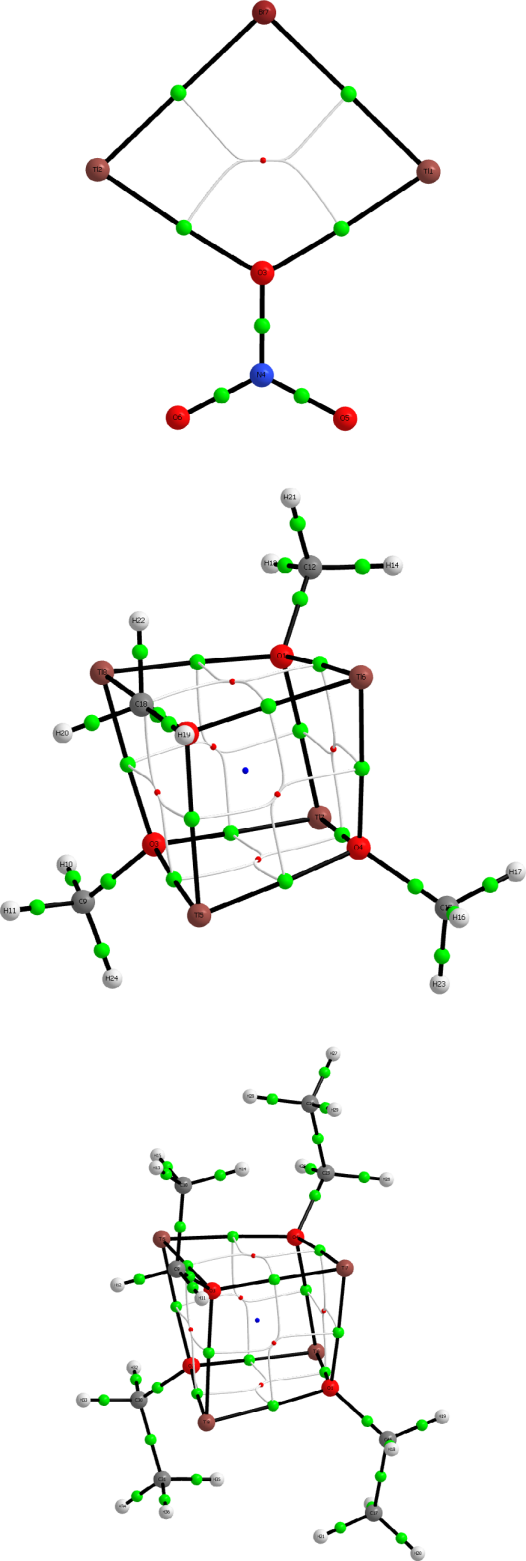
Atoms‐in‐molecules (AIM) images of the Tl(I) systems in order from Table 4 at MP2 level including bond (green), ring (red), and cage (blue) critical points, as well as ring‐to‐bond critical point paths highlighting that covalent bonding only can be observed along the ring/cage edges

**FIGURE 9:**
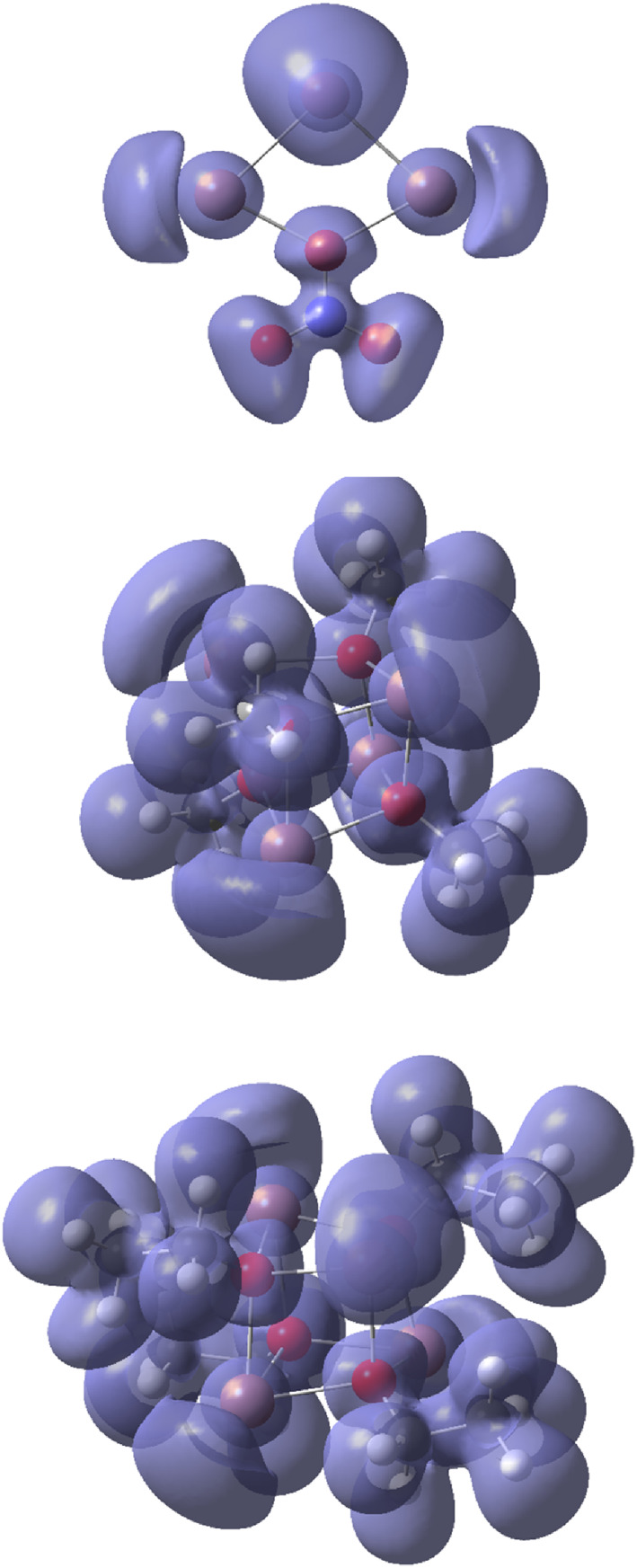
3D electron localization function (ELF) isosurfaces of the Tl(I) systems in order from Table 4 at MP2 level

The results from the subsequent AIM and ELF analyses essentially mimic the ones for the Pb(II) systems in the sense that there is no evidence for any direct metal–metal bonding in these molecular entities, see Figures 8 and 9.

Just as for the previously discussed lead(II) ring/cage systems, also the thallium(I) systems described in Figure 6 and Table 3 were exposed to an NCI and NEDA analysis. The results are given in Table S3 and Figure S6. The NCI isosurfaces communicate the same message as for the analogous lead(II) systems, where there is a significant covalency in the Tl(I)—OR bonds. Just as indicated in the spectral analyses by Maroni and Spiro, the NEDA decomposition into energy contributions show that the charge transfer is weaker than in the lead(II) systems, but so are the electrostatic contributions. Overall, the interaction energies are lower, but the covalent contribution is on a relative scale higher.

The main conclusion from the computational investigation of the M_2_^*m*+/−^ dimers, as well as from the molecular ring and cages systems of Pb(II) and Tl(I), is that the strong Raman‐spectroscopic features observed experimentally originate from a combination of the covalent character of the edge ring/cage M—X bonding together with the high symmetry of the molecular systems generating a cooperative effect in the vibrationbreathing modes. This combination offers a sufficient change in molecular polarizability in the totally symmetrical ring/cage vibration modes at low wavenumbers to generate a significant Raman activity, experimentally observed as strong, polarized peaks. Since the main electron density is accumulated on the heavy main‐group elements Pb(II) and Tl(I), it is natural to attribute the observed short metal–metal bond distances and characteristic spectral features to originate from the large electron density on the metal centres. However, as the above theoretical analysis shows, the actual reasons are less intuitive.

## Supporting information


**Appendix S1** Supporting information

## Data Availability

The data that support the findings of this study are available from the corresponding author upon reasonable request.
