# Supplementary material for: On closed‐shell interactions between heavy main‐group elements
Source: J Comput Chem. 2022 Sep 21;43(29):1985–96. doi: 10.1002/jcc.26999 (PMC9825979; doi:10.1002/jcc.26999)
Supplement: Supplementary file 1 — Appendix S1 Supporting information [file JCC-43-1985-s001.doc]

**Supplementary Information**

###### On Closed-Shell Interactions between Heavy Main-Group Elements

Lars Kloo

Applied Physical Chemistry, Department of Chemistry, KTH Royal Institute of Technology,

S-100 44 Stockholm, Sweden

**Interaction Types**

Figure S1 shows the potential energy surfaces for all M2^m+/-^ systems, with the direct electrostatics retained and removed. Figure S2 shows the results from application of the models described in equations SE1-SE3 below.


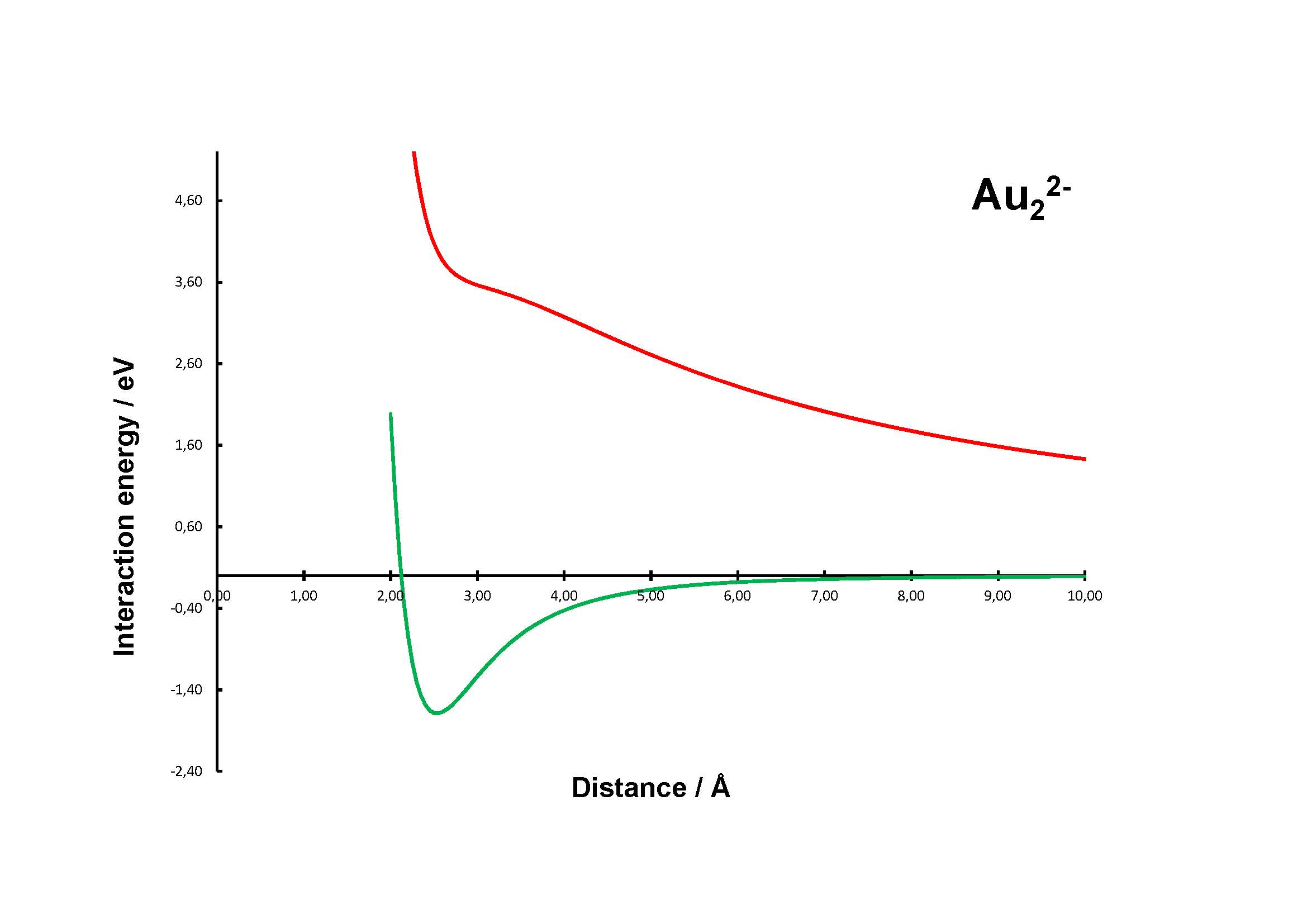

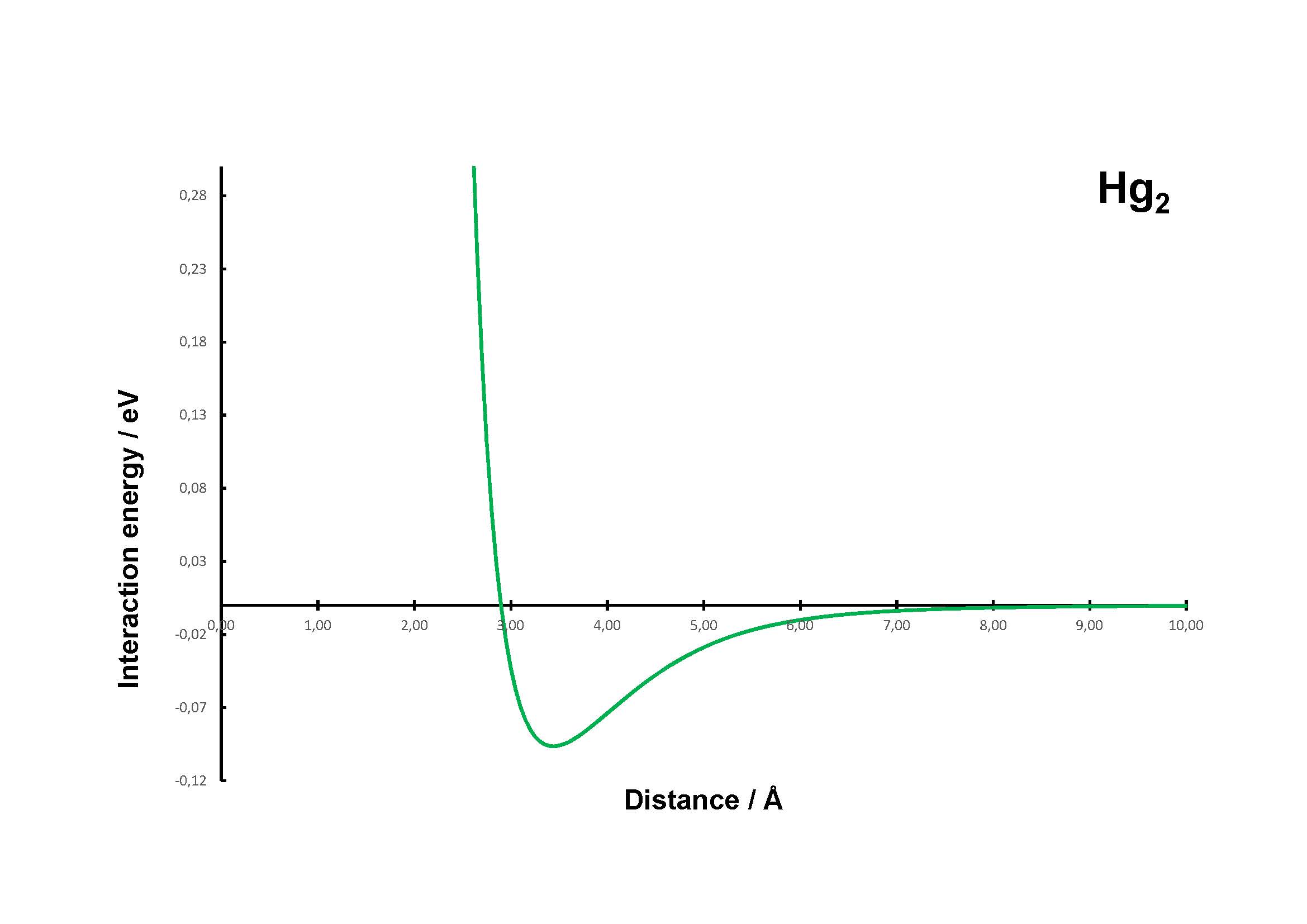


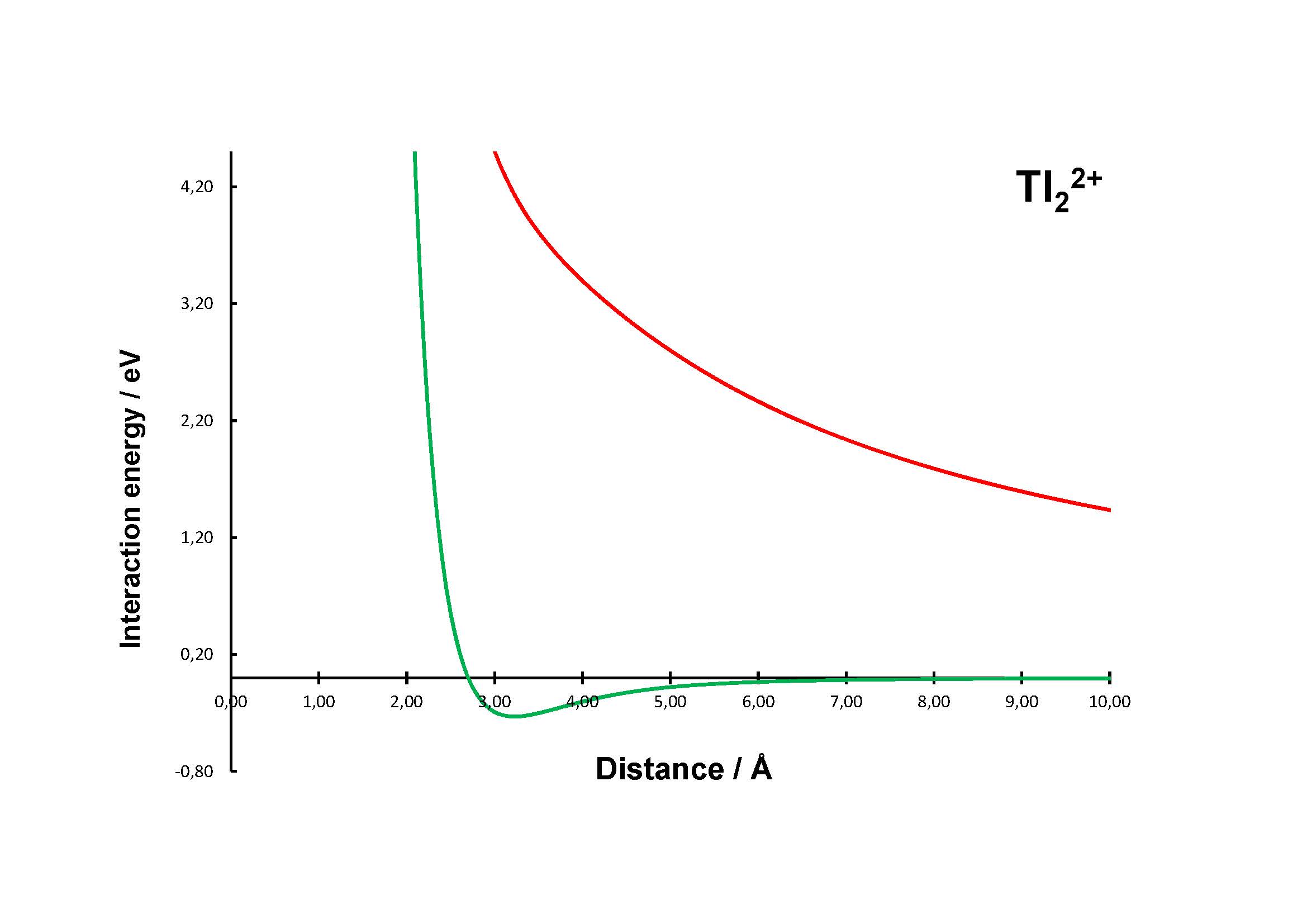

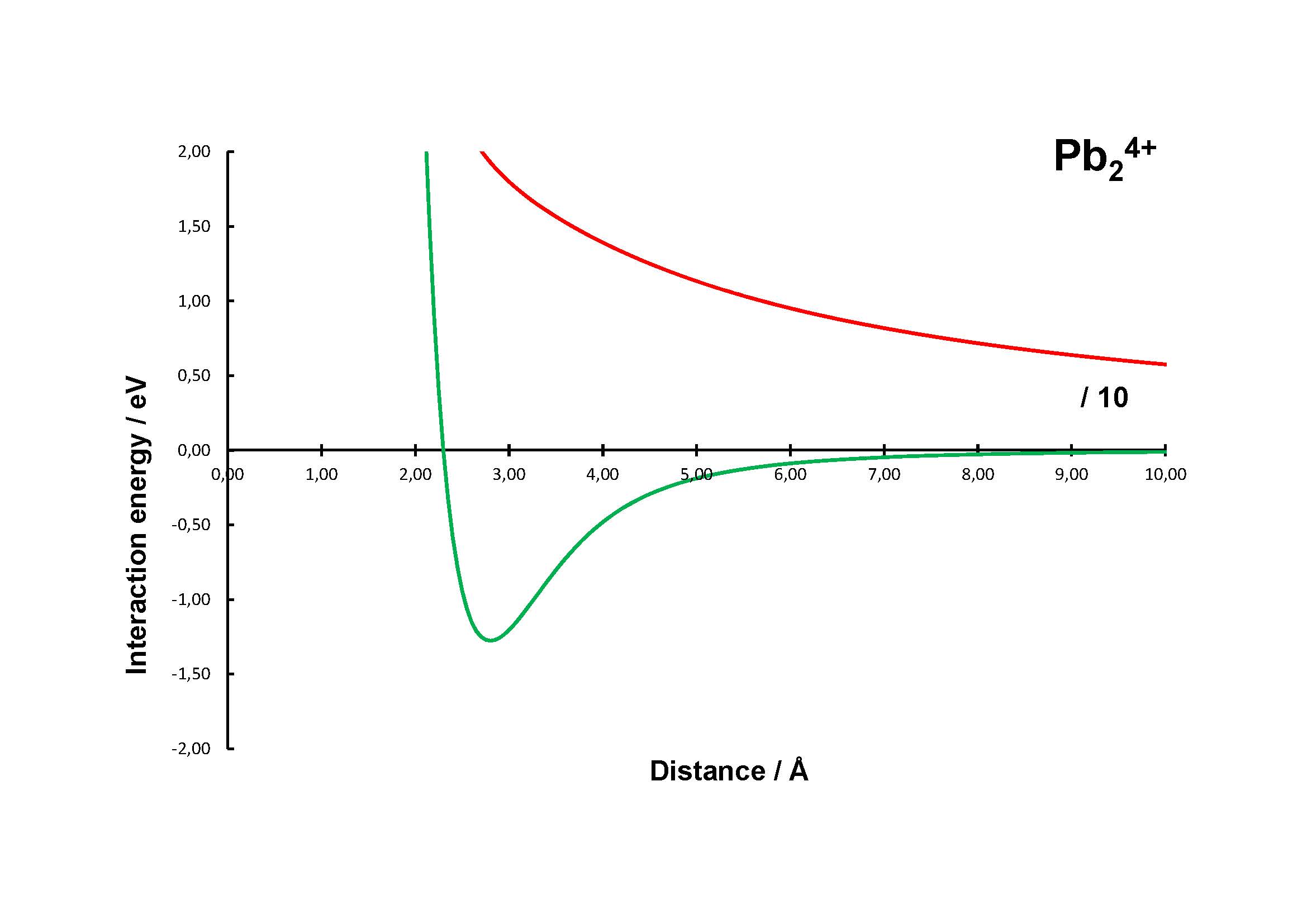


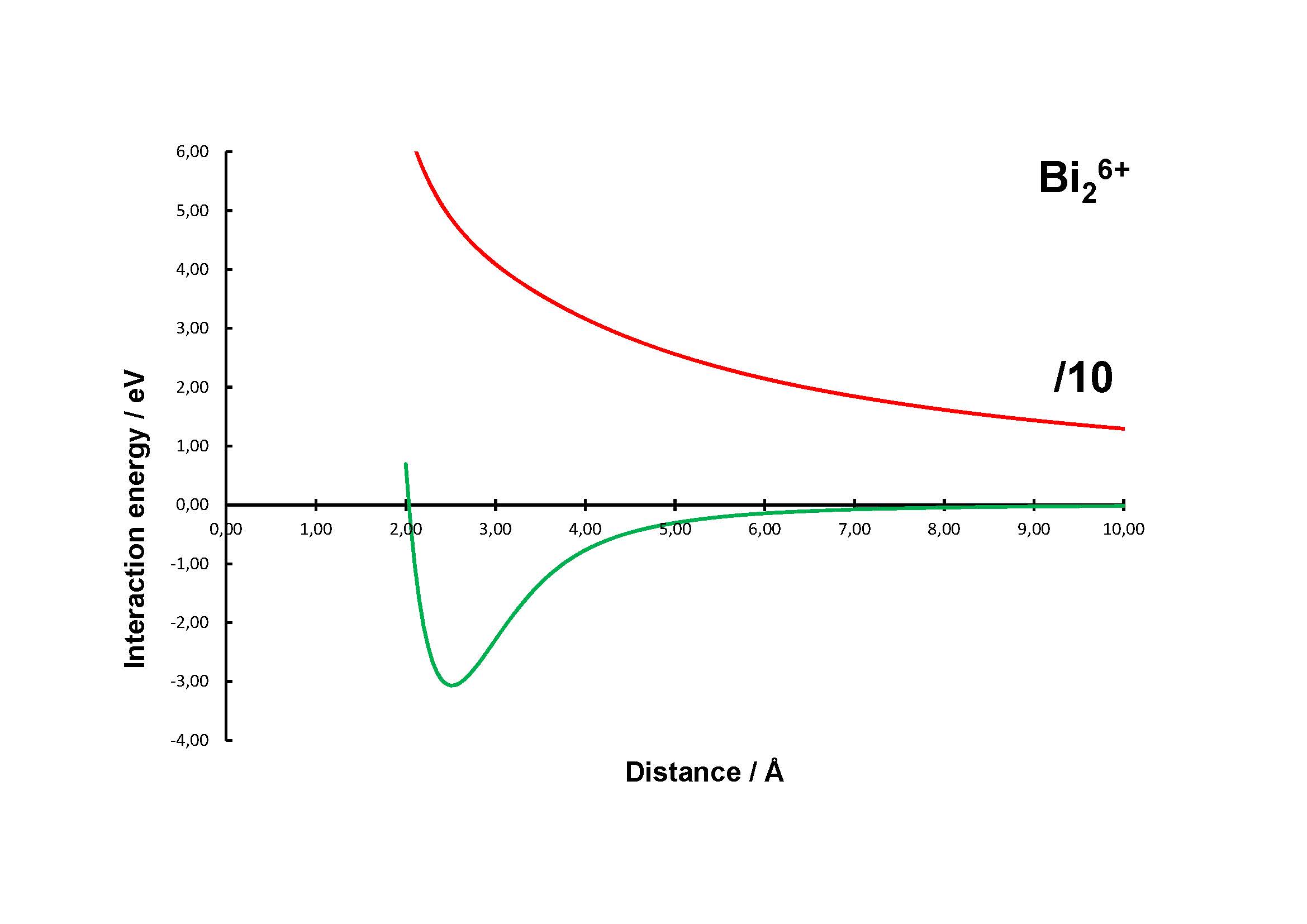


Figure S1. The PESs of all five representative systems at MP2- level for the binuclear systems including (red) and excluding (green) direct electrostatic repulsion.


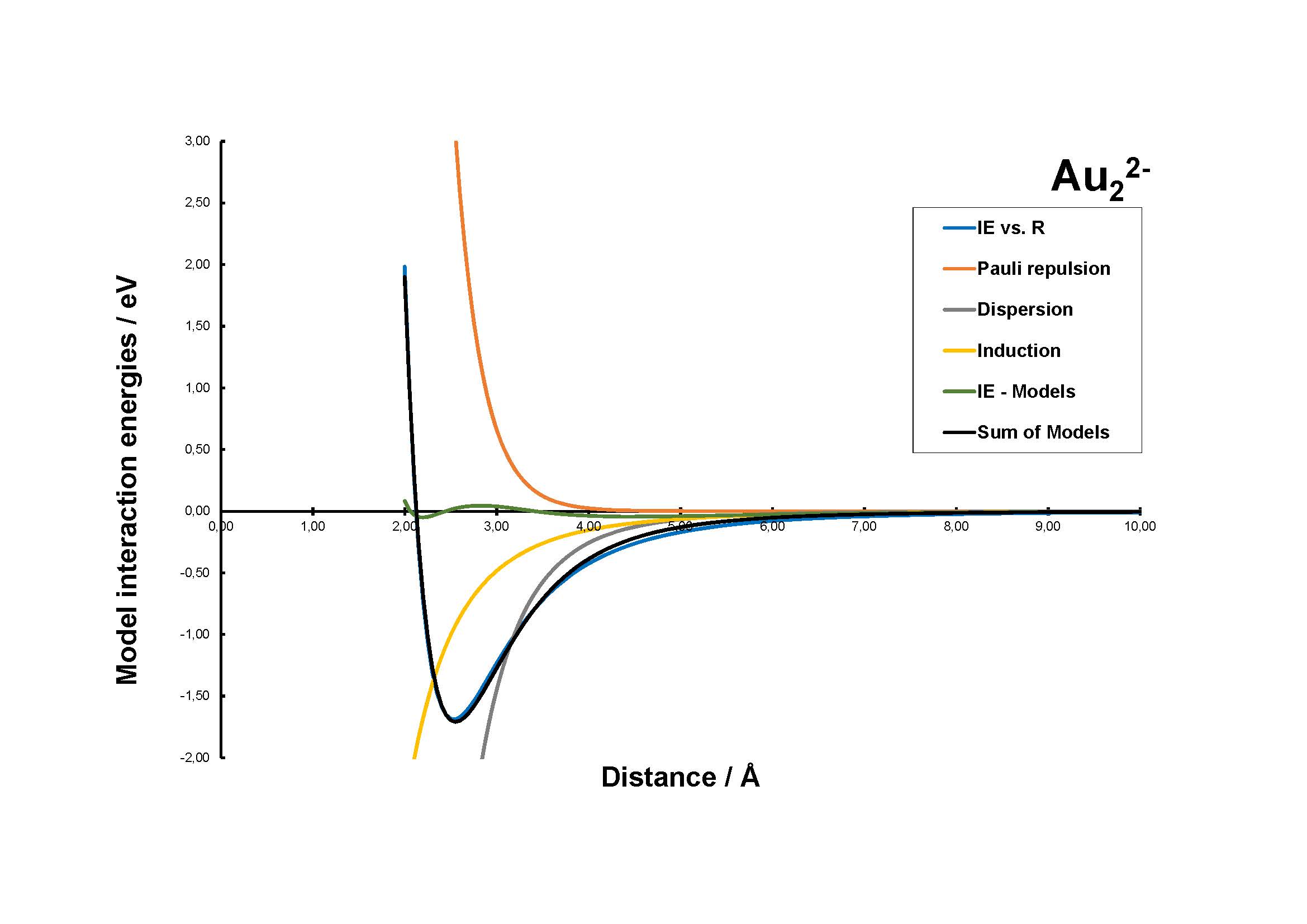

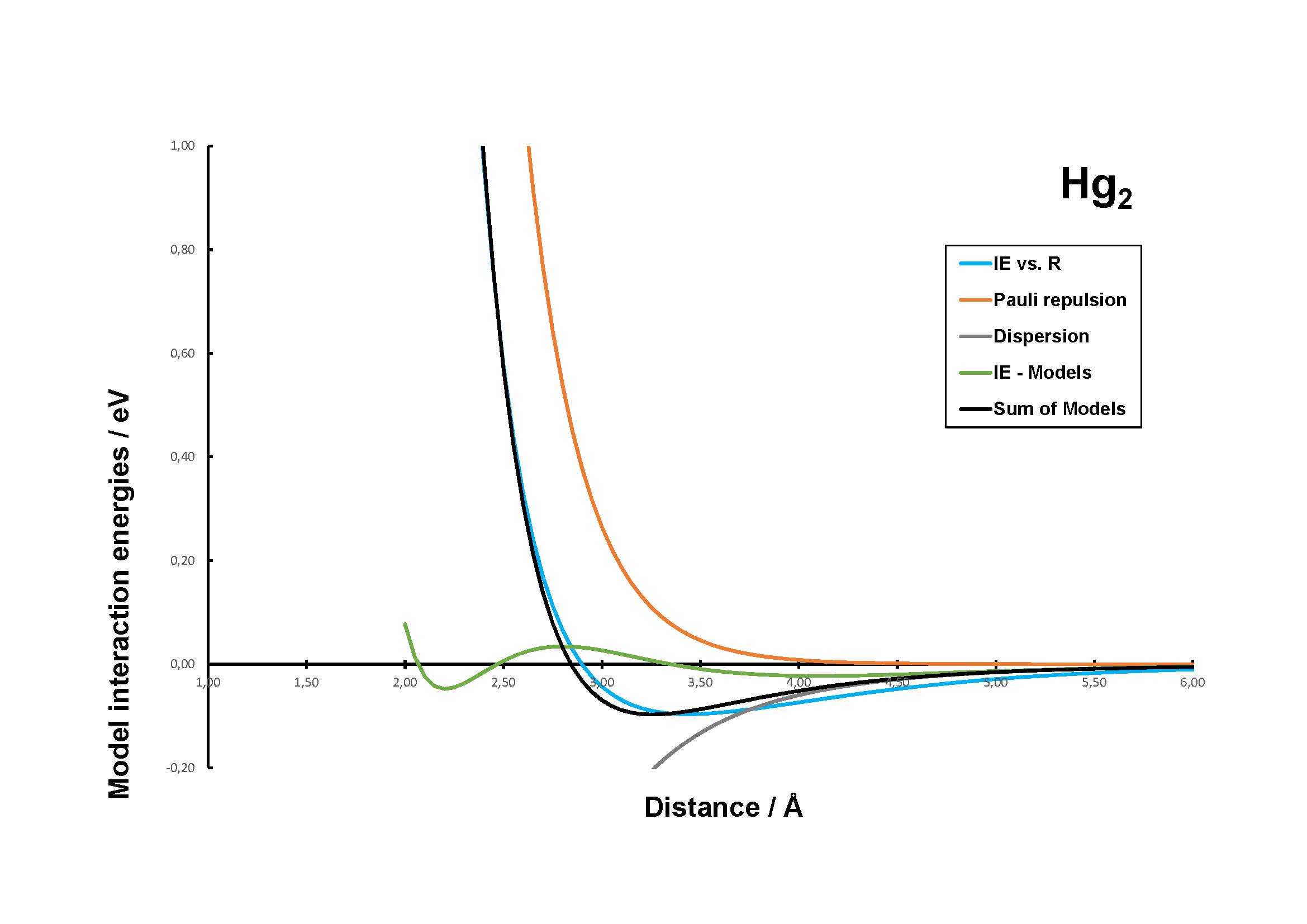


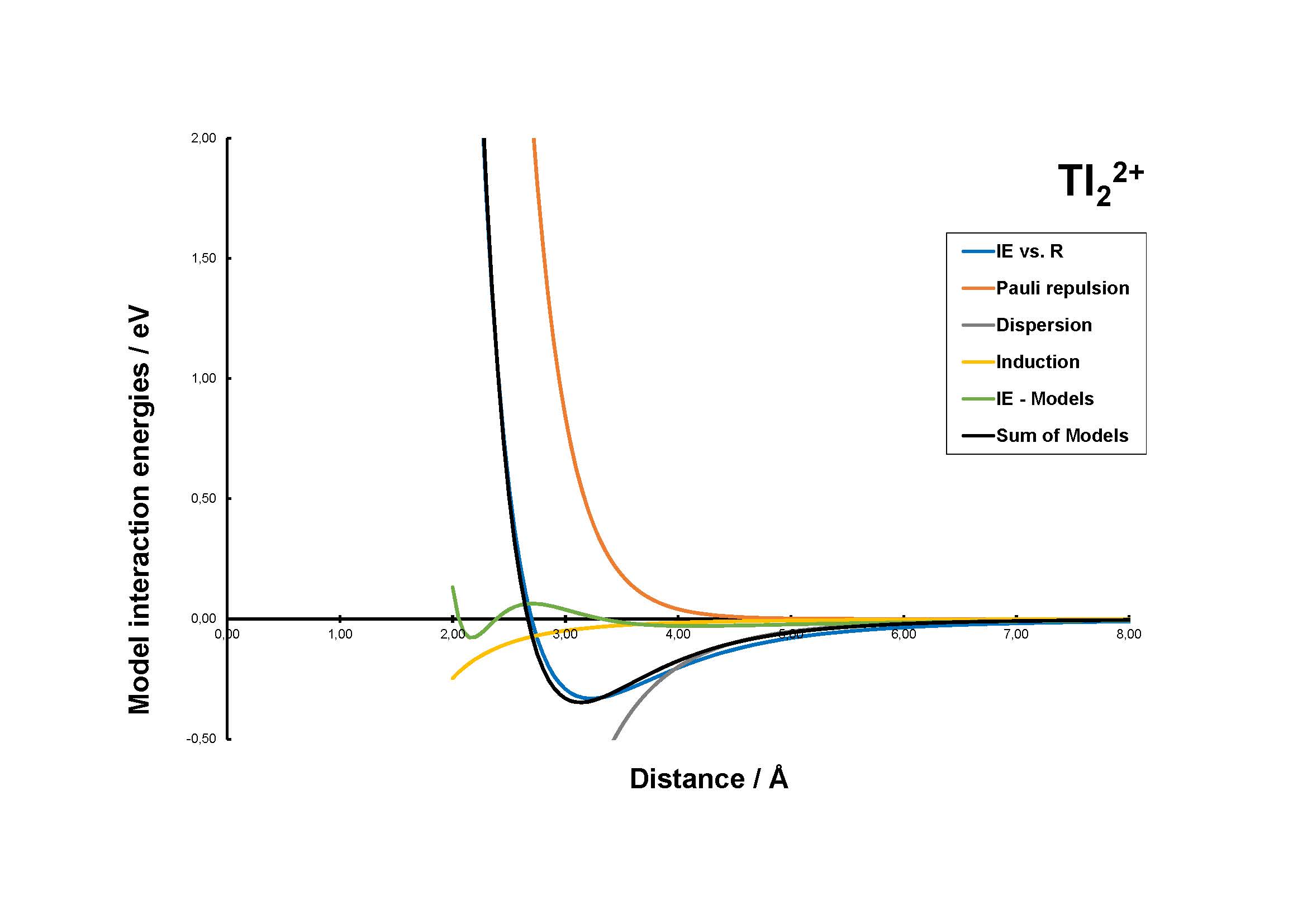

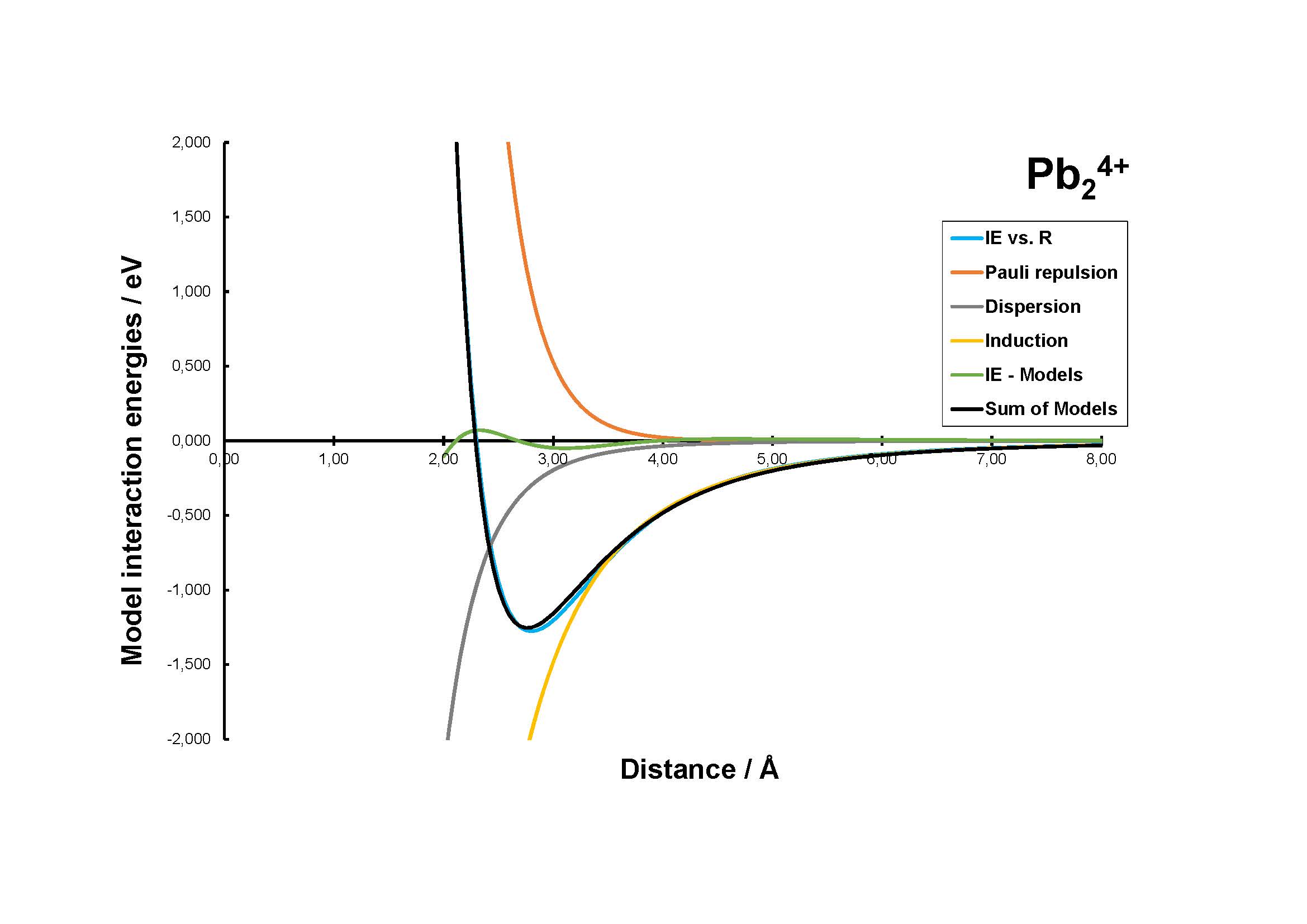


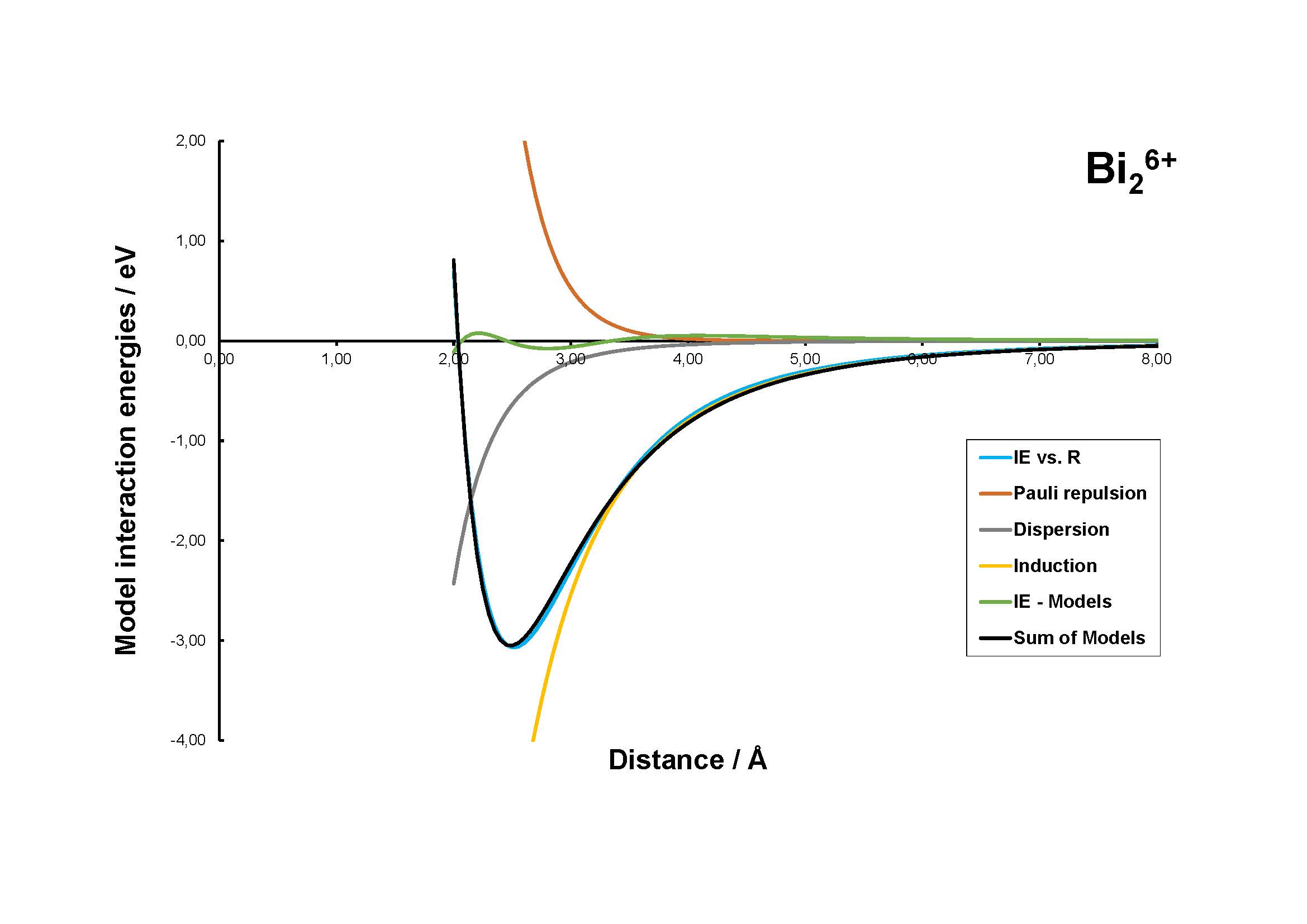


Figure S2. Models applied to the PESs at MP2- level for all five binuclear systems excluding direct electrostatic repulsion.

Figure S3 shows the dln(E)/dlnR relationship for all M2^m+/-^ systems, where E denotes the interaction energy and R the distance, arriving at 6 for Hg_2_ system and at 4 for all the charged M_2_ systems. This indicates a 1/R^6^ dependence of the interaction energy for the Hg_2_ system, suggesting dispersion to be the dominant type of interaction, and a 1/R^4^ dependence for the charged systems, instead indicating induction to be predominant.


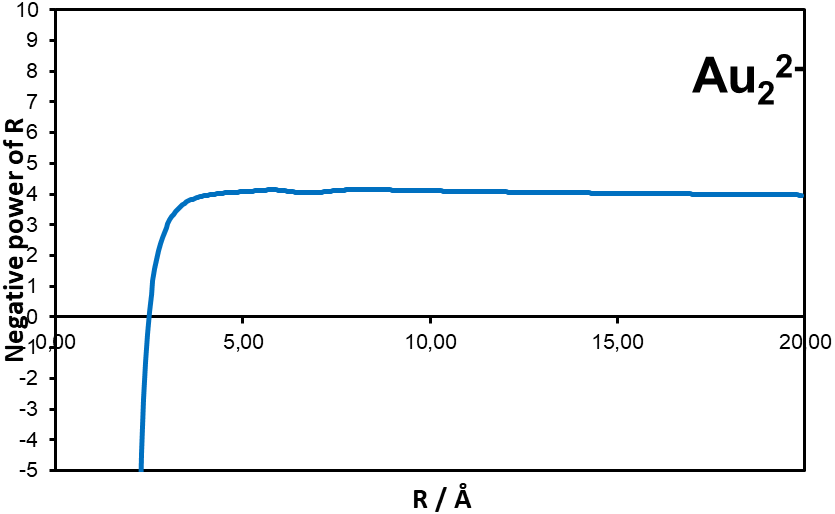

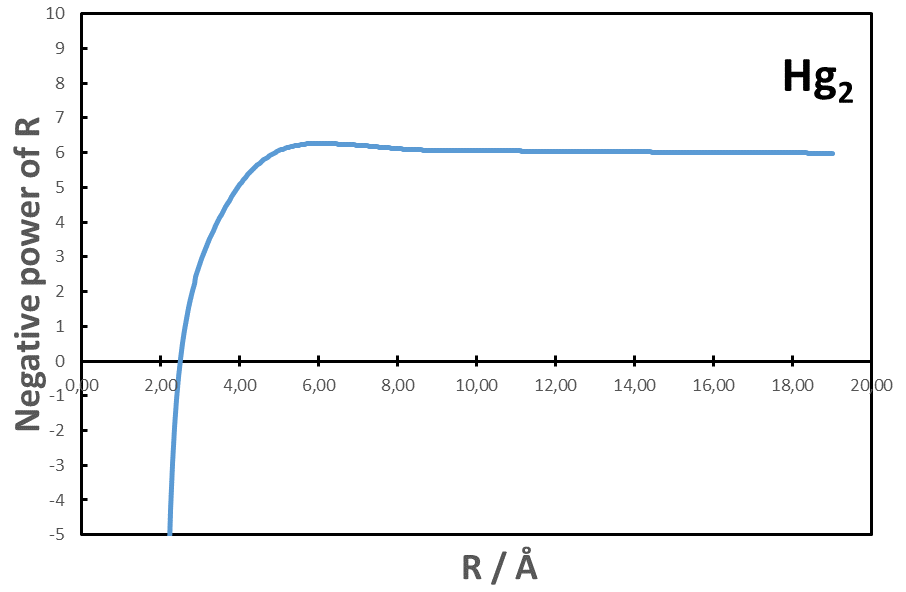


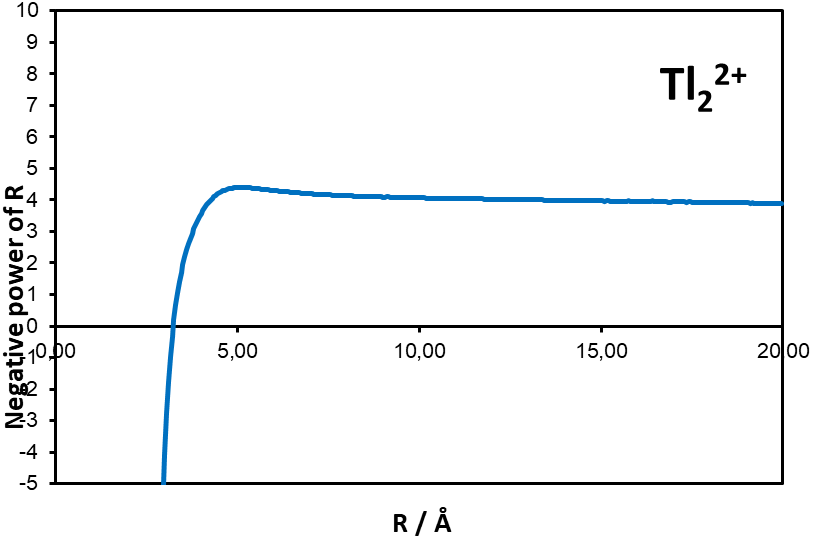

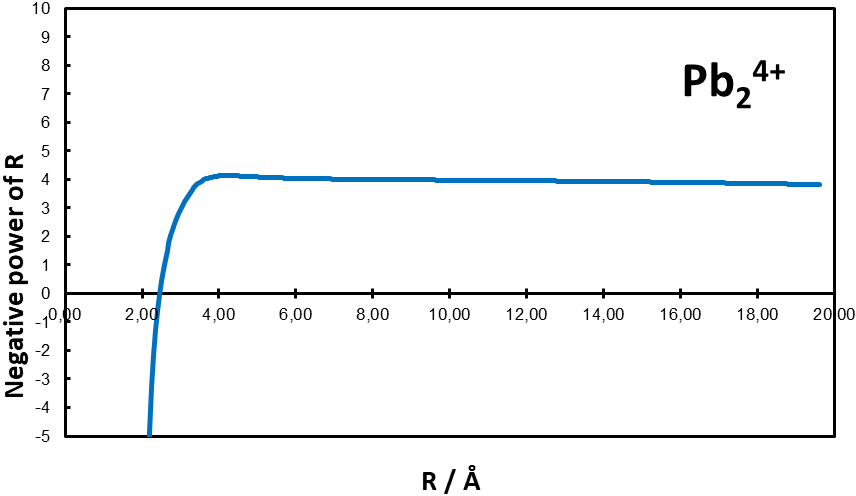


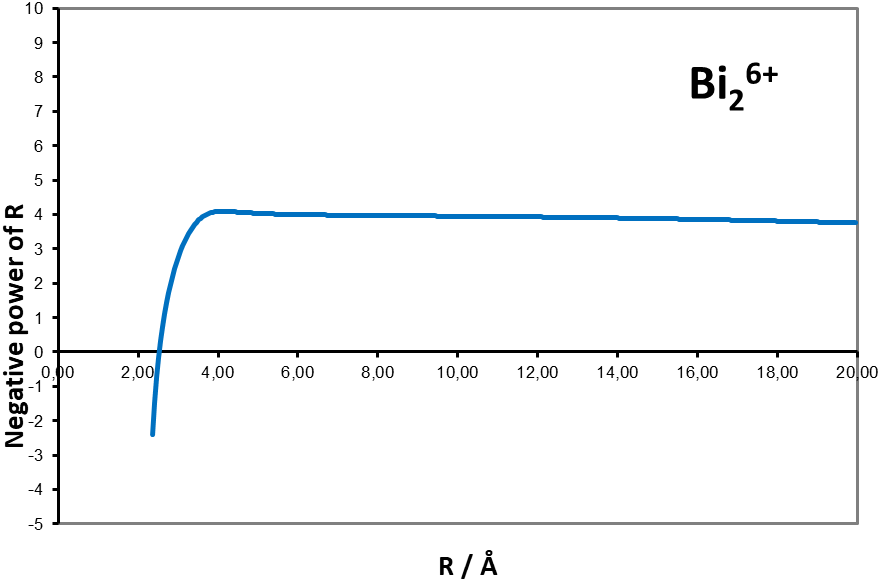


Figure S3. dln(E)/dln(R) for all five M_2_^m+/-^ systems representing the dominant power of R in the interactions

In order to gain a deeper insight into the interaction scheme in the five dimeric systems, simple models of different types can be applied to the PESs. By building complexity from the most simple and expected types of interactions, we will learn what is needed to model the PESs and thereby what types of interactions that, alongside direct electrostatic repulsion, will account for the ‘stickiness’ of the heavy, closed-shell atoms. Going from interaction types acting at shorter distances and then extending to those acting at longer distances, the following models were applied: 1, Pauli repulsion, Eq. SE1; 2, Dispersion following a 1/R^6^ relation, Eq. SE2; 3, Induction following a 1/R^4^ relation, Eq. SE3.

As can be noted in the formulation of Pauli repulsion in Eq. SE1, very minimized models will be applied in order to minimize model bias. The same philosophy was applied also to the subsequent models for dispersion and induction.

E_rep_ = A exp((-a R)/R), (SE1)

where A and a are obtained from the least-squares fit.

E_disp_ = -B exp(1/R^6^), and (SE2)

E_ind_ = -C exp(1/R^4^), (SE3)

where, again, the pre-exponential parameters B and C were obtained from the minimization of square differences. All models were simultaneously fitted to the computed PESs using a least-squares approach. As noted in the results, these very slim model are sufficient to model the predominant interactions in the pure, non-bridged binuclear systems. Although also other models, such as Morse interaction to phenomenologically represent the exponential distance dependence of orbital interaction, were considered but found to be unnecessary to include in the PES modelling.

**NCI and NEDA Analyses**

The iso-surfaces of an NCI investigation are shown in Figure S4. The iso-surfaces displayed have a contour value of 0.5 au.


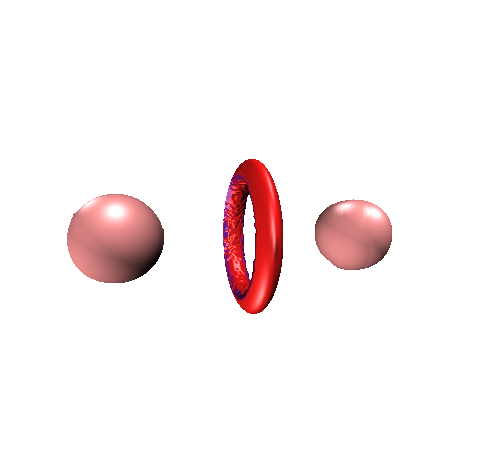

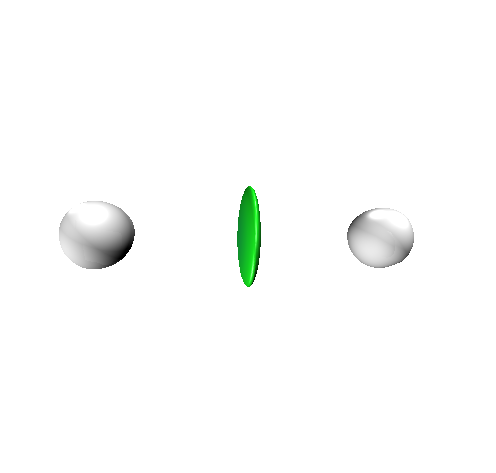

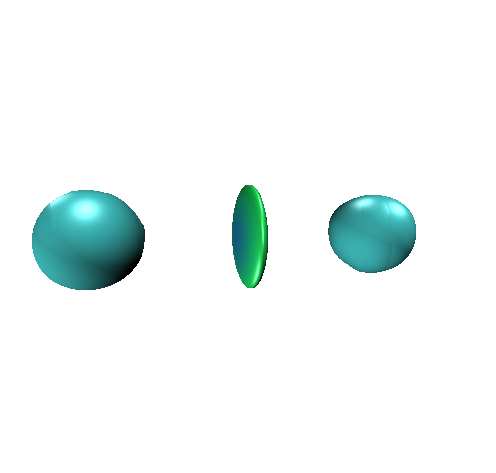


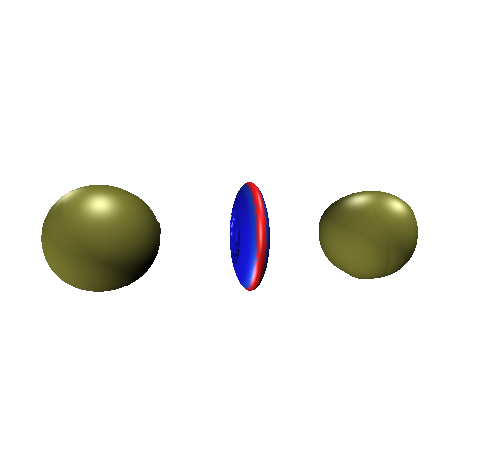

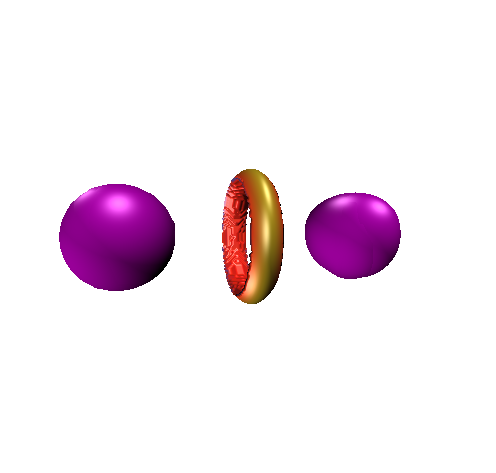


Figure S4. NCI iso-surfaces of the dimeric M_2_^m+/-^ systems. Red or light brown colours represent predominantly steric (repulsive) interactions, green van der Waals-type of interactions and blue interactions involving an accumulation of electron density. In order, Au, Hg, Tl, Pb and Bi.

The NEDA method partitions the interaction energy into a selection of contributions, and the most relevant to this study are given in Table S2. ES is the static Coulombic interaction, POL is the induced polarization energy and CT is the charge-transfer interaction between filled and non-filled orbitals of the two fragments (here represented by the separate metal atoms/ions). The total interaction energy E(tot) will also depend on the quantum-mechanical exchange energy, self-polarization energy and deformation energy in the NEDA scheme (not included in Table S1), and therefore the total interaction energy will not simply result as a sum of the three components given in Table S1.

Table S1. Results from a NEDA analysis of the M_2_^m+/-^ systems

| **Dimer** | **ES / kcal/mol** | **POL kcal/mol** | **CT / kcal/mol** | **E(tot) / kcal/mol** |
| --- | --- | --- | --- | --- |
| Au_2_^2-^ | +31,98 | -26.96 | -48.77 | +75.56 |
| Hg_2_ | -3.75 | -2.22 | -2.94 | -0.96 |
| Tl_2_^2+^ | +101.56 | -17.99 | -10.85 | +97.03 |
| Pb_2_^4+^ | +471.22 | -59.02 | -26.34 | +444.78 |
| Bi_2_^6+^ | +1190.88 | -134.60 | -52.10 | +1121.88 |

The corresponding NCI and NEDA results for the Pb^2+^ and Tl^+^ ring and cage systems follow below.


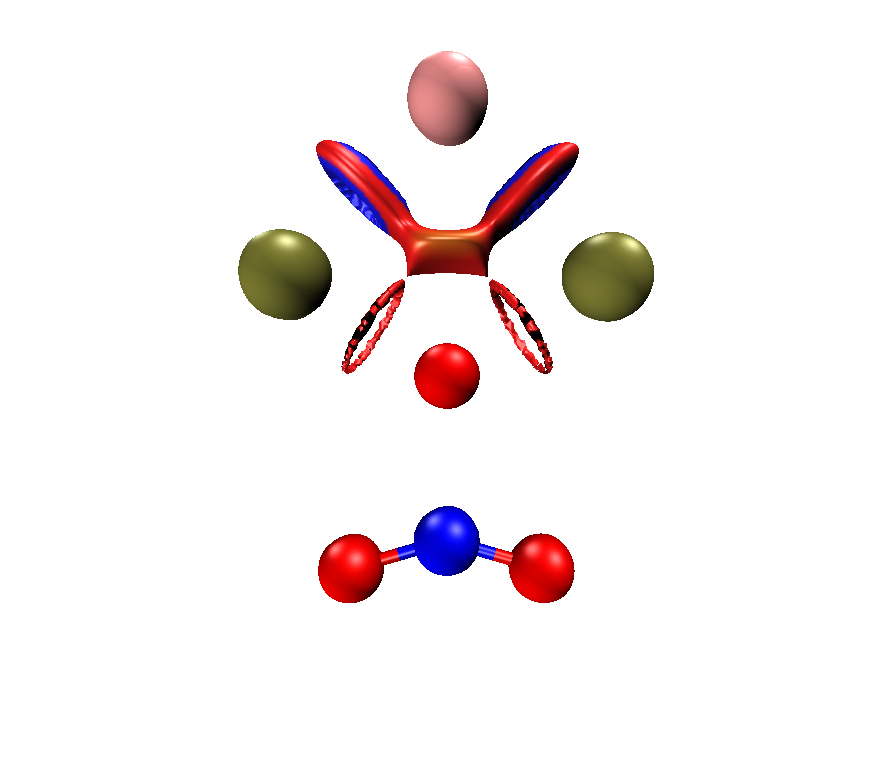


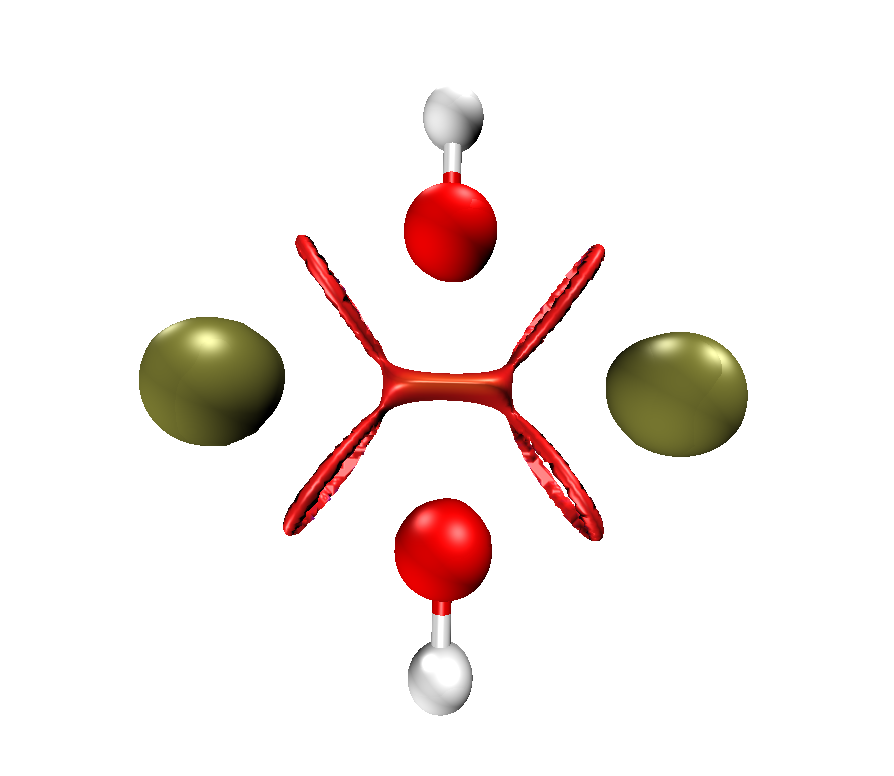


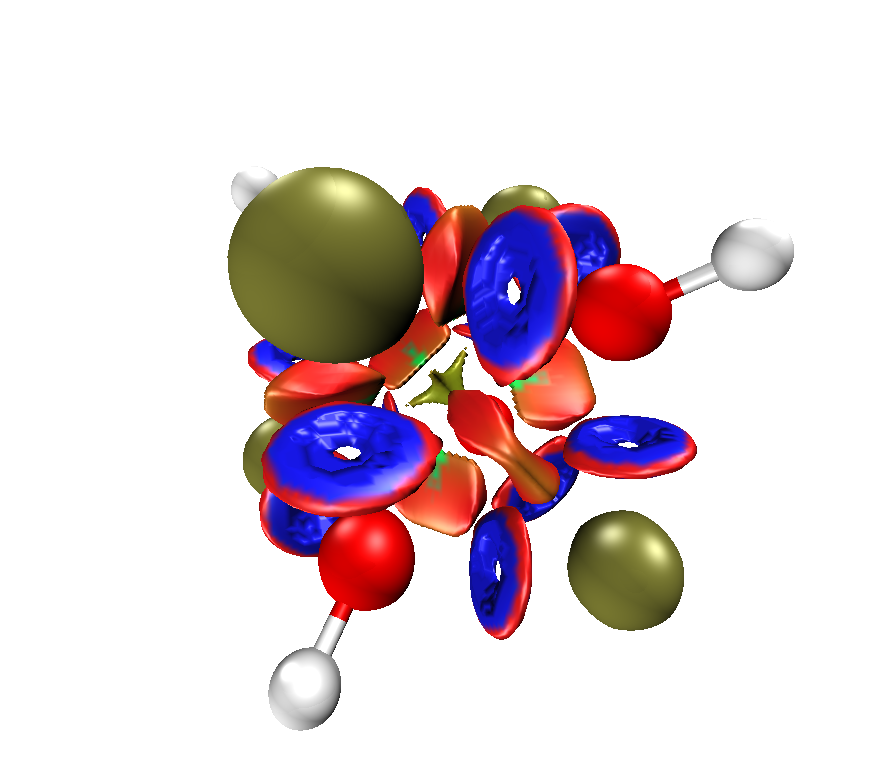


Figure S5. NCI iso-surfaces of the ring/cage systems containing Pb^2+^. Red or light brown colours represent predominantly steric (repulsive) interactions, green van der Waals-type of interactions and blue interactions involving an accumulation of electron density.

Table S2. Resuts from a NEDA analysis of the lead(II) ring/cage systems

| **Dimer** | **ES / kcal/mol** | **POL kcal/mol** | **CT / kcal/mol** | **E(tot) / kcal/mol** |
| --- | --- | --- | --- | --- |
| Pb_2_BrNO_3_^2+^ | -680.54 | -356.79 | -347.11 | -661.76 |
| Pb_2_(OH)_2_^2+^ | -820.44 | -297.57 | -266.58 | -711.45 |
| Pb_4_(OH)_4_^4+^ | -1422.63 | -469.51 | -477.14 | -1190.95 |


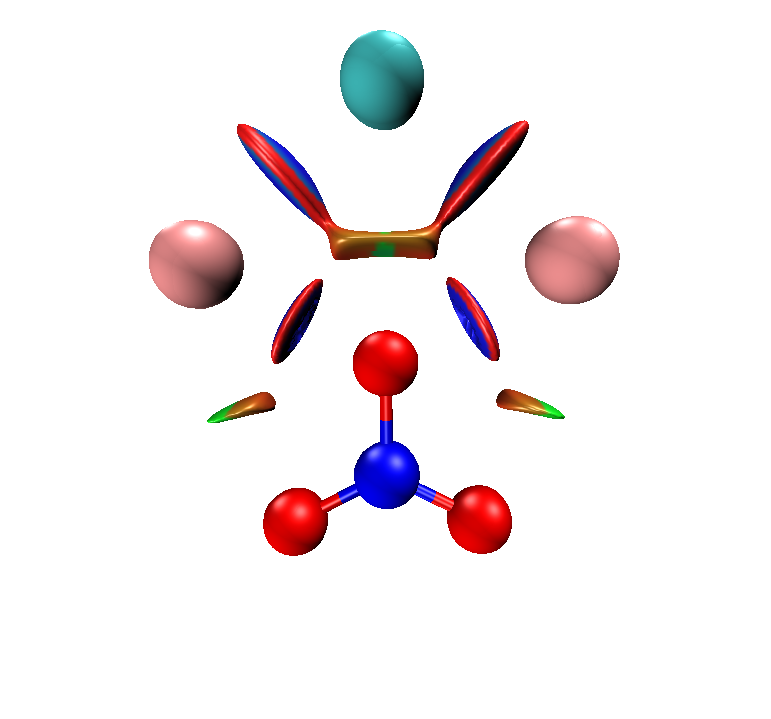


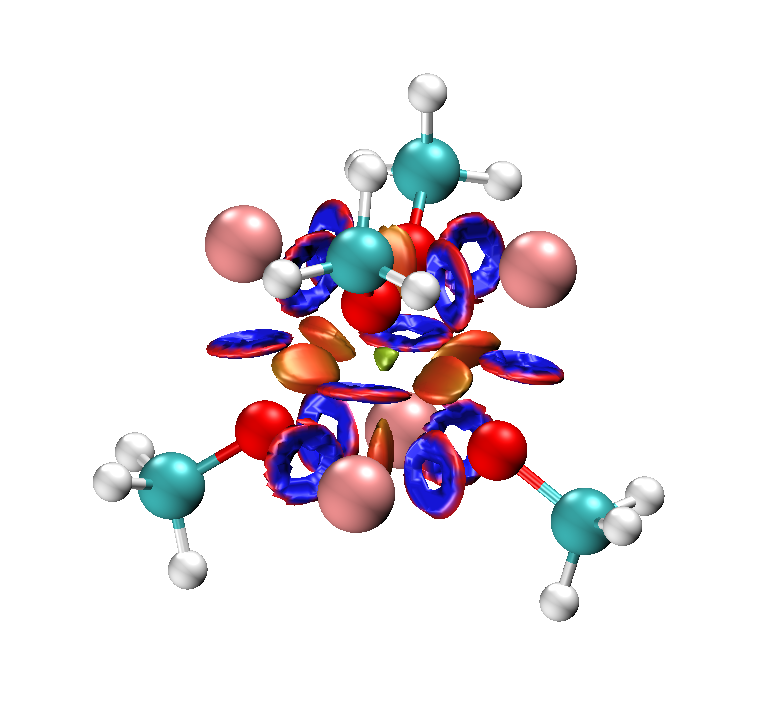


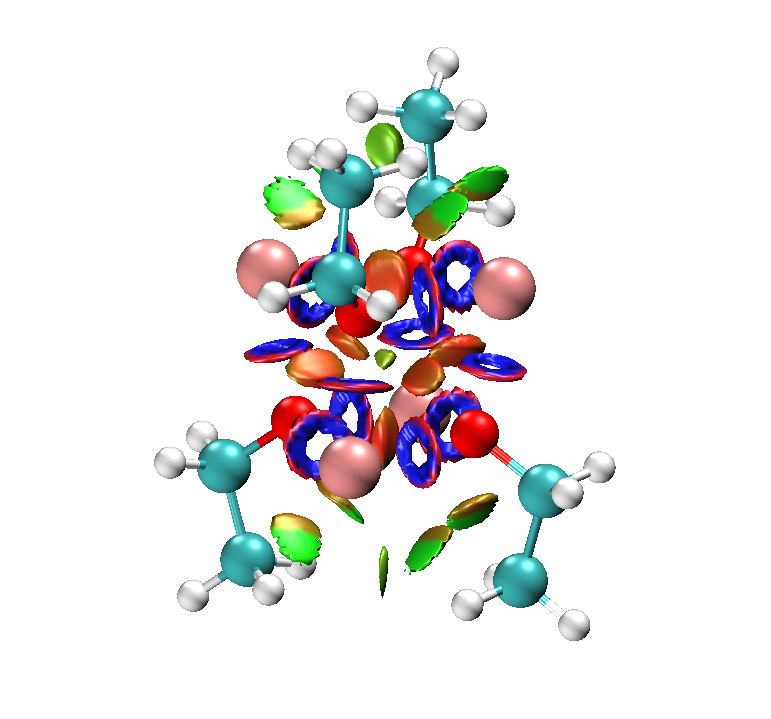


Figure S6. NCI iso-surfaces of the ring/cage systems containing Tl^+^. Red or light brown colours represent predominantly steric (repulsive) interactions, green van der Waals-type of interactions and blue interactions involving an accumulation of electron density.

Table S3. Results from a NEDA analysis of the lead(II) ring/cage systems

| **Dimer** | **ES / kcal/mol** | **POL kcal/mol** | **CT / kcal/mol** | **E(tot) / kcal/mol** |
| --- | --- | --- | --- | --- |
| Tl_2_BrNO_3_ | -336.40 | -110.00 | -120.40 | -293.58 |
| Tl_4_(OMe)4 | -1072.22 | -656.64 | -348.89 | -744.50 |
| Tl_4_(OEt)_4_ | -873.44 | -513.90 | -324.55 | -744.06 |
